# Supplementary material for: Patient participation in Delphi surveys to develop core outcome sets: systematic review
Source: BMJ Open. 2021 Sep 1;11(9):e051066. doi: 10.1136/bmjopen-2021-051066 (PMC8413947; doi:10.1136/bmjopen-2021-051066)
Supplement: Supplementary data [file bmjopen-2021-051066supp001.pdf]

Patient participation in Delphi surveys to develop core outcome sets: systematic review

Authors: Barrington H.J.[1], Young B.[1] & Williamson P.R.[1]

Author affiliations: [1]University of Liverpool, Liverpool, U.K.

Supplementary tables

Table 1a – Study characteristics (professional participants)

Professional recruitment source & approach[a]	
**Professional recruitment source**	**n (%)**
Professional organisation	49 (70%)
Publication authors (including Cochrane authors)	22 (31%)
Research study	13(19%)
Research group / consortium / CTU groups (including Cochrane group)	32 (46%)
Steering group members / contacts / University contacts	14 (20%)
Treatment centres	15 (21%)
Snowball sampling	25 (36%)
Other[b]	See below
Not reported	8
**Professional recruitment approach**	**n (%)**
Email invitation	50 (91%)
Postal invitation	4 (7%)
Handed invitation	4 (7%)
Newsletter / webpage	5 (9%)
Unclear	3
Not reported	20
**Participant characteristics reported**	
**Professional participants**	**n (%)**
Clinical experience	20 (26%)
Research experience	9 (12%)[c]
Gender	24 (31%)[d]
Age	21 (27%)e
Ethnicity	4 (5%)c
Education	3 (4%)f

Footnotes

[a] More than one recruitment source could be used

[b] Other included journal editorial groups (9), through informal mailing lists (n=2), members of steering committee (n=2), conference / conference special interest group (n=4) email discussion group / special interest group (n=4), research funding organisation (n = 2), audit participant (n=1)

[c]Includes 2 studies where characteristic reported collectively on research experience and ethnicity for PE and LE

[d]Includes 9 studies where characteristic reported collectively on gender for professionals and patients

[e]Includes 5 studies where characteristic reported collectively for professionals and patients

[f]Includes 1 study where characteristic reported collectively for professionals and patients

Table 1b - Study development and design characteristics of the Delphi studies

Study design & development characteristics	
**Number of rounds where patients participated**	**n (%)**
1	13 (17%)
2	28 (36%)
3	37 (47%)
**Number of stakeholder participant categories**	**n (%)**
2	31 (40%)
3	20 (26%)
4	16 (21%)
5	10 (13%)
6	1 (1%)
**Number of reported items per round**	**Descriptive statistics[a]**
Round 1 (n=71)	Median = 46, Min = 9, Max = 130
Round 2 (n=53)	Median = 49, Min = 8, Max = 130
Round 3 (n=28)	Median = 37, Min = 7, Max = 114

Footnote

[a]excluding not reported, n/a, unclear

Table 2a – Delphi characteristics rounds 2 and 3

Scoring System Rounds 2 &3		
**Scoring system**	**Round 2 n (%)**	**Round 3 n (%)**
1-9 / 1-10[a]	52 (85%)	26 (77%)
0-4/1-4 / 1-5	4 (7%)	3 (9%)
9/10/12 most important outcomes	2 (3%)[b]	1 (3%)
Yes/no/don't know or agree/disagree/unsure	2 (3%)	1 (3%)
Yes/no/include in COS & Essential and recommended outcomes	n/a	3 (9%)
Domain inner core, middle ring, outer ring	1 (2%)	n/a
Not reported	2	1
Unclear	2	2
n/a patients only in 1 round	13	13
n/a only 2 rounds	0	28
**Feedback**		
**Feedback type Round 3**		**n (%)**
All stakeholder groups combined		7 (28%)

2

Stakeholder groups reported separately	9 (36%)
Own stakeholder group	1 (4%)
Each stakeholder group & all stakeholder groups combined	3 (12%)
Own stakeholder group & all stakeholder groups combined	3 (12%)
SWAT	2 (8%)
Not reported	6
N/a only 2 rounds	28
N/a patients only took part in one round	13
Unclear	6

Footnotes

[a]Only two studies used 1-10

[b]Caregivers scored differently to patients in one of these studies – patients used score cards

Supplementary Figure 1 - Preferred Reporting Items for Systematic Reviews and Meta-Analyses flowchart of identification of eligible studies from the COMET database. Data were extracted from the COS systematic reviews

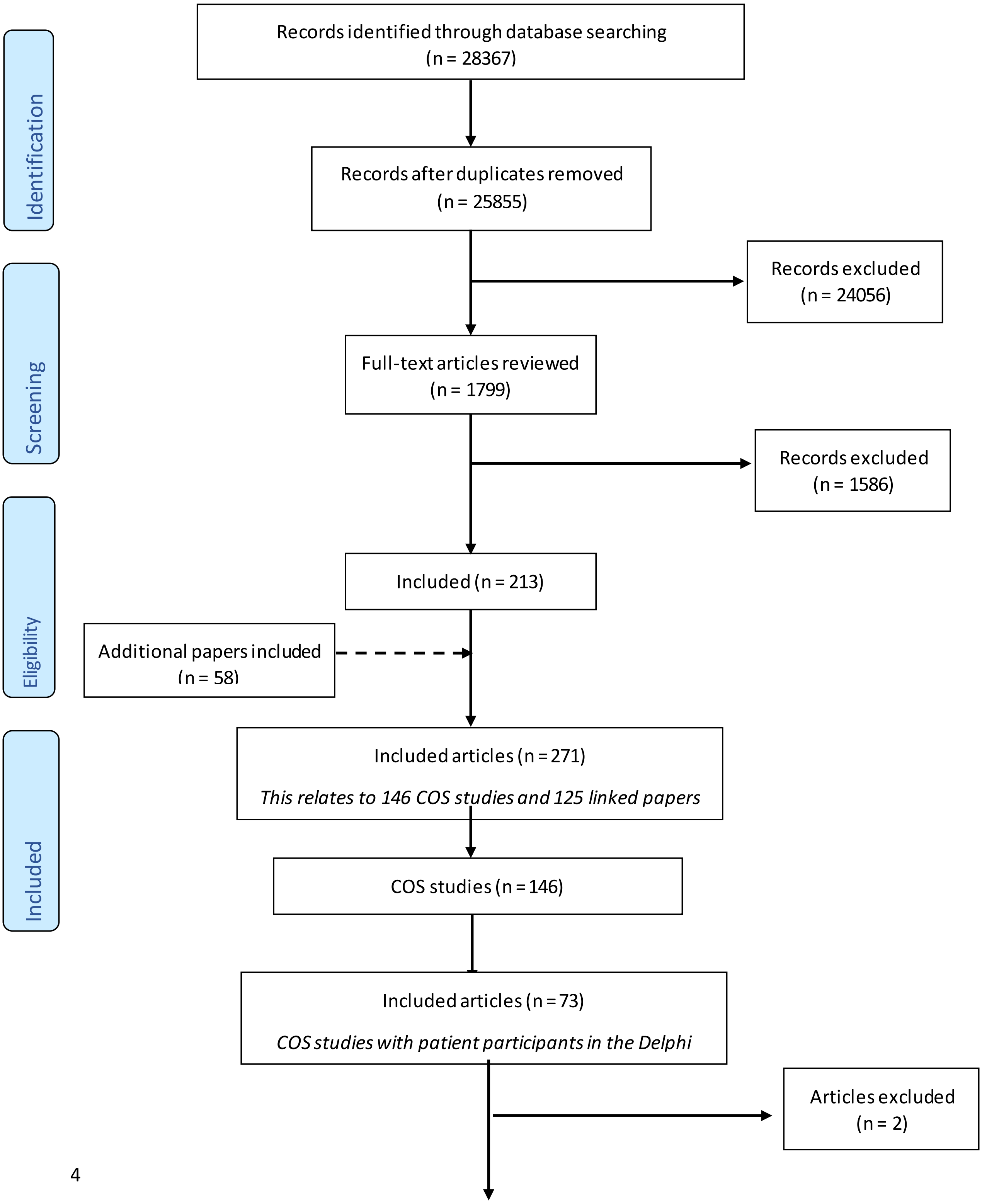

Included articles (n = 71)

COS studies with more than one patient participant in the Delphi
