# Supplementary material for: Patient participation in Delphi surveys to develop core outcome sets: systematic review
Source: BMJ Open. 2021 Sep 1;11(9):e051066. doi: 10.1136/bmjopen-2021-051066 (PMC8413947; doi:10.1136/bmjopen-2021-051066)
Supplement: Author's manuscript [file bmjopen-2021-051066.draft_revisions.pdf]

BMJ Open

BMJ Open is committed to open peer review. As part of this commitment we make the peer review history of every article we publish publicly available.

When an article is published we post the peer reviewers' comments and the authors' responses online. We also post the versions of the paper that were used during peer review. These are the versions that the peer review comments apply to.

The versions of the paper that follow are the versions that were submitted during the peer review process. They are not the versions of record or the final published versions. They should not be cited or distributed as the published version of this manuscript.

BMJ Open is an open access journal and the full, final, typeset and author-corrected version of record of the manuscript is available on our site with no access controls, subscription charges or pay-per-view fees (http://bmjopen.bmj.com).

If you have any questions on BMJ Open's open peer review process please email info.bmjopen@bmj.com

BMJ Open

Patient participation in Delphi surveys to develop core outcome sets: systematic review

Journal:	*BMJ Open*
Manuscript ID	bmjopen-2021-051066
Article Type:	Original research
Date Submitted by the Author:	09-Mar-2021

Complete List of Authors:	Bagley, Heather; University of Liverpool, Health Data Science<br>Young, Bridget; University of Liverpool, Department of Psychological Sciences<br>Williamson, Paula; The University of Liverpool, Biostatistics

Keywords:	STATISTICS & RESEARCH METHODS, GENERAL MEDICINE (see Internal Medicine), SURGERY

SCHOLARONE™
Manuscripts

I, the Submitting Author has the right to grant and does grant on behalf of all authors of the Work (as defined in the below author licence), an exclusive licence and/or a non-exclusive licence for contributions from authors who are: i) UK Crown employees; ii) where BMJ has agreed a CC-BY licence shall apply, and/or iii) in accordance with the terms applicable for US Federal Government officers or employees acting as part of their official duties; on a worldwide, perpetual, irrevocable, royalty-free basis to BMJ Publishing Group Ltd ("BMJ") its licensees and where the relevant Journal is co-owned by BMJ to the co-owners of the Journal, to publish the Work in this journal and any other BMJ products and to exploit all rights, as set out in our licence.

The Submitting Author accepts and understands that any supply made under these terms is made by BMJ to the Submitting Author unless you are acting as an employee on behalf of your employer or a postgraduate student of an affiliated institution which is paying any applicable article publishing charge ("APC") for Open Access articles. Where the Submitting Author wishes to make the Work available on an Open Access basis (and intends to pay the relevant APC), the terms of reuse of such Open Access shall be governed by a Creative Commons licence – details of these licences and which Creative Commons licence will apply to this Work are set out in our licence referred to above.

Other than as permitted in any relevant BMJ Author's Self Archiving Policies, I confirm this Work has not been accepted for publication elsewhere, is not being considered for publication elsewhere and does not duplicate material already published. I confirm all authors consent to publication of this Work and authorise the granting of this licence.

For peer review only

Patient participation in Delphi surveys to develop core outcome sets: systematic review

Authors: Barrington H.J.[1], Young B.[1] & Williamson P.R.[1]

Author affiliations: [1]University of Liverpool, Liverpool, U.K.

Author email addresses: heather.bagley@liverpool.ac.uk, byoung@liverpool.ac.uk, prw@liverpool.ac.uk.

Correspondence to: Heather Barrington: heather.bagley@liverpool.ac.uk

Keywords: core outcome set; systematic review; patient participation; Delphi surveys

Word count: 3432

For peer review only

Abstract

Objectives: To describe the design and conduct of core outcome set (COS) studies that have included patients as participants, exploring how study characteristics might impact their response rates.

Design: Systematic review of COS studies published between 2015 and 2019 that included more than one patient, carer or representative as participants (hereafter referred to as patients for brevity) in scoring outcomes in a Delphi.

Results: There were variations in the design and conduct of COS studies that included patients in the Delphi process, including, differing: scoring and feedback systems, approaches to recruiting patients, length of time between rounds, use of reminders, incentives, patient and public involvement and piloting. Minimal reporting of participant characteristics and a lack of translation of Delphi surveys into local languages were found. Additionally, there were indications that studies which recruited patients through treatment centres had higher round 2 response rates than studies recruiting through patient organisations.

Conclusions: Variability was striking in how COS Delphi surveys were designed and conducted to include patient participants and other stakeholders. Future research is needed to explore what motivates patients to take part in COS studies and what factors influence COS developer recruitment strategies. Improved reporting would increase knowledge of how methods affect patient participation in COS Delphi studies.

Article summary

Strengths and limitations of this study

- This is the first systematic review of patient participation in Delphi surveys for core outcome set development.

For peer review only - http://bmjopen.bmj.com/site/about/guidelines.xhtml

- This comprehensive review explored both study characteristics and recruitment and retention rates amongst patients.
- The findings are limited by reporting issues in the reviewed studies, especially on recruitment and few studies reported how many individuals received the initial invitation to participate.
- Other reporting issues, including on patient and public involvement, limit the conclusions that can be drawn from this review.

Background

Patients and health care professionals need evidence about what treatments work best to inform their health care decisions. The results of clinical trials are, however, often difficult to compare due to a lack of standardisation in the outcomes measured for the same health condition and challenges with reporting bias[1]. In addition, including the perspectives of patients on what outcomes matter to them is crucial[2] Core outcome sets (COS) are a potential solution to these problems, providing standardised sets of outcomes, developed and agreed upon by key stakeholders, including patients.

COS are developed through iterative consensus building processes. Commonly a systematic review and sometimes qualitative interviews with patients are used to explore patients' views on outcomes and generate a long list of potential outcomes. These outcomes are then taken forward into a consensus process, most gathering views through a Delphi survey and ratifying these results at a consensus meeting to agree a COS[3]. Delphi participants are invited to score outcomes in several survey 'rounds', considering the feedback of other expert groups as part of the process. Delphi surveys lend themselves to e-surveys and as such can be widely distributed, however, like other questionnaires, these surveys are prone to low response rates[4].

Patient participation in COS studies has increased over recent years, with Gorst et al[5] reporting 77% of published COS studies included patients or their representatives. While this paper focusses largely on patient participation in COS, it is important to distinguish between this and patient involvement in COS studies. When patients participate, they are contributing data on which outcomes to prioritise, for example scoring outcomes in Delphi studies. When patients are involved in COS studies they are helping to design and oversee the COS study from a patient / public perspective. There are several challenges in including patient participants in COS and indeed there are indications some COS developers 'problematise' patient participation[6], highlighting for example, the tendency for patients to rate many outcomes highly. Biggane et al[7] found that patients without prior experience of Delphi surveys expressed difficulty understanding both the purpose of the COS and particular aspects of the surveys. Young and Bagley[8] called for further research exploring how patient input is currently being sought in COS studies and to understand more about the challenges of including and engaging patients in COS development.

To the authors' knowledge no review of patient participation in COS Delphi studies has previously been published. We have undertaken a systematic review of recent COS studies that have included patients in their COS Delphi, to describe how these studies have been designed and conducted and how study characteristics might impact patient participant response rates. By identifying challenges in recruiting and retaining patients in COS studies this review aimed to inform strategies to optimise the participation of patients in future COS studies.

For peer review only - http://bmjopen.bmj.com/site/about/guidelines.xhtml

Methods

The protocol is available at: www.comet-initiative.org/Studies/Details/1824

Study selection

Inclusion criteria Eligible COS studies were those identifying outcomes for use in research, published between 1st January 2015 and 31st December 2019, and including more than one patient, carer or their representative as a participant (hereafter referred to as patients for brevity) in scoring outcomes in a Delphi as part of the process.

Identification of relevant studies: Studies were identified through the COMET Initiative database. How studies are identified for inclusion in this database has previously been described[3, 5, 9-13]. Briefly, eligible studies for the database were those that employed methodology to gain consensus as to which outcome domains or outcomes should be measured in clinical trials or other forms of health research. Any studies that described the update of an existing COS are included in the database as linked papers to the original COS. Eligible studies are added to the database as they are identified, and an annual systematic review of these is published to ensure the database is kept current.

Studies meeting the criteria for our review were selected from the aforementioned database. Where authors referred the reader to the protocol in the methods section of their article, these protocols were also reviewed. Studies reporting updates to COS studies that were already in the COMET database were not included in the current review.

Data extraction

A data extraction template was developed including the following domains:

- **Study scope** – Health area; the population; intervention type; location (participating countries).
- **Study development and design** – Methods to explore patients' views on outcomes; survey language and translation, participant groups represented; number of rounds; number of outcomes in each round; reported PPI and piloting; scoring and feedback systems used; use of reminders and other incentives; recruitment sources and methods.
- **Study conduct and results** – Reporting of participant characteristics; response rates in each round by participant group; ratio professionals (PE, i.e. participants not providing a patient perspective, such as health care professionals and researchers): patients in round 1.

Some studies had included patients and other stakeholders earlier in their COS, for example in generating a list of outcomes, and authors sometimes referred to these as 'rounds'. Only rounds relating to the scoring of outcomes were included in this review. Data extraction was undertaken by one person (HB) with checking of certain technical aspects, such as the methods of feedback, by a second person (PW).

Data analysis

In addition to describing how studies had been designed and conducted, we were keen to explore whether participation rates were linked with other study design variables. We anticipated, for example, that more personalised recruitment approaches or the use of incentives or reminders might impact response rates and that steps to enhance the design of surveys such as patient and public involvement (PPI) and piloting might also impact patient participant responses. Additionally,

For peer review only - http://bmjopen.bmj.com/site/about/guidelines.xhtml

we wished to explore whether the recruitment source used in a study influenced patient participation. The two most commonly used recruitment sources were patient organisations and treatment centres, therefore these were chosen for comparison. As several studies used both these sources we also explored their combined influence on participation.

Patient and public involvement

Patients and the public were not involved in the design, conduct, or reporting of this review of previously published data.

Results

The PRISMA diagram for the review in presented in Supplementary Figure 1. From a total of 146 COS studies published between 2015 and 2019, 73 COS studies were initially identified as eligible, however 2 of these were subsequently excluded as only one patient had participated. Of the 71 included COS studies, 66 reported on a single core outcome set. The remaining 5 studies reported on a total of 12 COS. For example, one article by Hall et al[14] reported on three COS for three different interventions in tinnitus. Patients could complete any or all of these Delphi surveys so recruitment and retention data for each of these COS studies could be different. After discussion it was decided to treat each COS individually. Of the five articles which reported on more than one COS, two each reported on three COS, and three articles each reported on two COS. In total, therefore 78 COS studies are included in this review. In thirteen of the COS studies, patients participated in only one round of scoring in the Delphi.

Study scope

Table 1 illustrates the scope of the included studies. The COS studies represented a broad range of health areas, with pregnancy and childbirth (14%, n =11) and cancer (12%, n = 9) being the most common. Whilst the COS were predominantly developed for adults (58%, n =45), 14% (n=11) were for children. Most COS were developed for any intervention (63%, n = 49). The median number of countries participating in the COS studies was 16 (in 18 studies the number of countries was either not reported or unclear), maximum 73, and 13% (n=10) were conducted in a single country. Where data was given for numbers of countries from which the patient participants were drawn, the maximum number of countries was 21.

Table 1 around here

Study characteristics

The variation in study characteristics can be seen in Table 2. In preparation for the Delphi study, the most common method used to explore patients' views on outcomes was by interview (n = 20, 26%).

Thirty six percent of studies (n = 28) described piloting the Delphi, whilst patient involvement in the study design or delivery was reported by 40% of studies (n=31), although the detail around the PPI and piloting was generally minimal.

Most COS studies were delivered electronically to patients (74%, n =39). Of the 51 studies that either reported on language used or where it was implicit in the description, o 20% (n=10) of studies described offering some form of translation of the study materials (including 3 COS studies in one article). Just over half the studies reported using reminders (56%, n=44). Only 8% (n=6) of studies described using incentives, 3 monetary incentives and 3 non-monetary (3 COS from the same article).

For peer review only - http://bmjopen.bmj.com/site/about/guidelines.xhtml

For peer review only

A range of recruitment sources were used to recruit patients and some studies used multiple sources. Patient organisations (62%, n = 43) and treatment centres (45%, n = 31) were the most common. The most common method of recruitment was by email (74%, n = 42). Supplementary Table 4a presents the data on professional recruitment sources and methods.

There was heterogeneity in reporting of patient participant characteristics. Only 10% (n = 8) reported on the patient socio-economic / educational status and only 9% (n = 7) on their ethnicity. Similarly, less than a third of studies reported on either patient experience of the condition, (e.g. length of experience) or an aspect of their treatment experience. Table 5a presents the reporting data on professional characteristics. Additional study design characteristics are presented in Supplementary Table 2a and study characteristics relating to professionals are in Table 2b

Table 2 around here

Table 3 presents the data on Delphi specific issues, including the duration of rounds, the scoring approaches in round 1 and feedback methods in round 2 (data for subsequent rounds are presented in Supplementary Table 3a) where both patients and professionals scored outcomes. Most studies did not report the duration of their rounds, however, of those that did, the majority reported 2-4 weeks duration per round. The majority of COS studies reported using a 1-9 scoring system (70%, n=52).

Feedback methods were explored for studies reporting more than 1 round. 48 studies reported on which stakeholder groups' feedback was presented to participants, for example, whether patient and professional feedback was presented separately for each group or combined. The most frequent approach was where results for different stakeholder groups were reported separately, (n=21, 44%). A range of feedback types were described by the 43 studies reporting on this, with some studies reporting use of two or more types of feedback. The most common type of feedback was the distribution of scores (65%, n =28); 10 studies (23% of those reporting) described providing either a mean or median only.

Table 3 around here

Table 4 shows the response rates per round. The recruitment sources of the 20 studies where patient response data for round 1 was reported were predominantly treatment centres (45%, n = 9). The median round 1 response rate for patients was 59% compared to 52% for professionals.

The median ratio of professionals to patients was 2.7 (n=61), although some studies reported more than twice as many patients as professionals (e.g. Potter[15]).

Participation rates for rounds 2 and 3 were calculated (excluding studies where non-respondents were invited from previous rounds). The median round 2 response rate for patients was 84% (n = 44), comparable to the professional respondents (median = 85%, n = 46). Response rates in round 3 were the same (91%) for both patients and professionals.

Table 5 explores potential associations between patient response rates, and PPI, Delphi piloting, reminders and methods of recruitment. There is limited reporting of data on these factors with no evidence of an effect of PPI, piloting and reminders on response rates but an indication that recruiting from treatment centres is better in terms of retention in round 2. Round 2 response rates for studies recruiting through treatment centres were higher (89%, n =6) than studies recruiting through patient organisations (77%, n = 20) and a combined treatment centre / patient organisation approach (77%, n = 11), although the numbers of studies were small, particularly for those recruiting through the treatment centre.

For peer review only - http://bmjopen.bmj.com/site/about/guidelines.xhtml

For peer review only

Discussion

This review has highlighted variations in the design and conduct of COS studies that included patients in the Delphi process, including differing: scoring and feedback systems, approaches to recruiting patients, lengths of time between rounds, and use of reminders, incentives, PPI and piloting. It has also identified potential challenges with the Delphi feedback approaches, minimal reporting of participant characteristics; the lack of translation of Delphi surveys into local languages and indicated that recruitment may be more of a challenge than retention. There were indications that studies which recruited patients through treatment centres had higher round 2 response rates than studies recruiting through patient organisations.

Williamson et al[16] recommend using qualitative research or consulting with key stakeholders, including patients, to help identify important outcomes and ensure that the language used to describe outcomes is meaningful for patients. Less than a third of studies used either of these two methods prior to undertaking their Delphi survey. Additionally, Williamson et al[1] suggest that piloting of the Delphi survey can also help the COS development team to refine their outcome labels and explanations, however, only around a third of studies report undertaking piloting. COS developers may be missing opportunities to improve the accessibility of their Delphi surveys. Better reporting of piloting would improve understanding of its impact.

Our review indicates that the 1-9 scoring system is most commonly used system in COS studies that include patients. Biggane et al[7] interviewed patients retrospectively about their experience of participating in a Delphi survey, noting that whilst there are statistical considerations influencing the choice of scoring scales, patients can have differing views on the scales used. Whilst some patients in their study preferred the 1-9 scoring scale, others struggled to use it, indicating the need for additional support and guidance. Given the high usage of the 9-point scoring method, further research is warranted to explore how patients and other participants experience, interpret and use this scoring system.

Providing feedback to participants on the scores of other participants in previous rounds is used to drive consensus between stakeholders in Delphi surveys, with stakeholders encouraged to consider the views of others before re-scoring an outcome. A study that compared providing feedback to participants only on the scores of their own peer group, versus providing feedback to participants on the scores from each of the stakeholder groups, found that seeing other groups' perspectives increased consensus[17]. Participants in a study by Fish et al[18]reported "trying to understand the importance of an outcome from the perspective of another participant", as one of the most common reasons for revising their scores between rounds, and this was especially the case for health care professionals. Whilst several studies in our review did not report on their feedback approach, nearly half of those that did report described providing reported on this did not provide feedback to participants by group, instead just presenting feedback from a participant's own stakeholder group or for all participants combined. In the absence of presenting each participant with feedback from each group consensus may not be so easily achieved across stakeholder groups[1]. Of note were two SWAT studies exploring feedback methods, indicating interest in finding the best feedback approach[19, 20]. One of these has been completed, finding that peer feedback reduced variability in scoring compared with combined feedback from multiple groups[19].

In addition to what feedback participants received about the scores of other participants, how feedback was presented also varied in the studies although most presented feedback as a distribution of scores and numerical frequencies. Of studies that reported on how feedback was presented, a fifth described only providing a summary statistic (a median or mean score). This is

For peer review only - http://bmjopen.bmj.com/site/about/guidelines.xhtml

potentially problematic as there are indications that participants do not understand the term median and that they have issues with fully understanding averages[21]. Fish[21] also found the patients in her study understood and liked seeing the percentage of participants rating each outcome as each of 1 to 9, and yet our review has found that around two thirds of studies did not provide such feedback. Further research is needed to explore the best ways to present feedback so that it is more easily understood.

The COS_STAD (Standards for core outcome set development) specifies that people with lived experience of the condition / intervention should be key stakeholders in the COS development process[22]. Our review explored the ratio of patient participants compared to professionals, finding that patients tended to be in the minority, although there are also examples of COS studies with higher rates of participation amongst patients (e.g. Potter et al[17]). Inclusivity in COS development is crucial to ensure that the outcomes selected in a COS are relevant and important for the diverse range of patients potentially affected by the COS. There have been calls for more inclusive research generally, further emphasised by the recent COVID 19 pandemic[23]. In the studies in our review, there was minimal reporting of patient ethnicity and socioeconomic status and the reasons for this warrants further exploration. Additionally, there was minimal reported use of translation meaning that COS completion is restricted to those with the relevant language skills, again limiting its inclusivity.

Given the need to ensure adequate stakeholder diversity and inclusion and the potential impact of attrition (overestimation of consensus if participants with minority perspectives drop out), it is important to explore response rates in all rounds of the COS studies. There are indications that recruiting stakeholder participants into COS studies can be challenging, however, once recruited, retention was quite high for most studies. This echoes findings from Delphi studies in other areas[4]. Retrospective interviews with patient participants in COS Delphi studies have highlighted key areas of concern for them and provided some initial insights on their motivation to participate[7]. However, further research is needed that explores patients' motivation to take part soon after the recruitment decision to inform the development of future recruitment resources.

Young & Bagley[8] described the potential benefits that PPI could bring to the COS development process. Less than half of the studies in this review reported undertaking PPI. Those studies that did report PPI provided scant details. The few that provided more detailed reports will help future COS developers plan for PPI (e.g. Smith[24] & Crudgington [25]). PPI may help with several aspects of a COS study, including recruitment and retention, for example by improving the accessibility of the study. Improving the reporting of PPI, for example, by following the GRIPP2 checklist[26], would enable the impact of PPI on recruitment and retention to be more accurately investigated.

We aimed to explore how study characteristics such as PPI, piloting, reminders, recruitment methods and sources influenced the participation of patients. The reporting of recruitment in the reviewed studies was complex and sometimes unclear. Our comparison of recruitment sources and response rates was limited due to problems with reporting. However, studies using treatment centres as a source for recruitment appeared to have higher round 2 response rates. This echoes previous findings [21] indicating lower attrition amongst patient participants recruited via treatment centres compared to those recruited through patient organisations and social media. This warrants further research.

This study is limited by omissions in reporting about the design and delivery of studies. Recent guidance about COS development and reporting[27] and guidance on PPI reporting[26] may improve the description of COS studies in the future. We are planning to interview COS developers to explore

For peer review only - http://bmjopen.bmj.com/site/about/guidelines.xhtml

their perspectives on the design of COS Delphi studies, including the use of patient facing resources to recruit and retain patients in a Delphi survey and materials to support their participation. We will work closely with a PPI panel to review these materials, alongside the findings of this current review and the future findings from interviews with COS developers, to enhance the accessibility, ease of use and appeal of the materials.

Conclusion

This study has explored the participation of patients in COS studies. Variability was striking in how COS Delphi surveys were designed and conducted to include patient participants and other stakeholders. Future research would be useful to explore what motivates patients to take part in COS studies and what factors influence recruitment strategies used by COS developers. Reporting needs to be improved to increase knowledge of how methods affect patient participation, in particular reporting response rates and denominators for all rounds by stakeholder group, more detailed descriptions of PPI, piloting, recruitment methods and sources.

References

1. Williamson, P., et al., *The COMET Handbook: version 1.0*. 2017: BioMed Central.
2. Berglas, S., et al., *Patients' perspectives can be integrated in health technology assessments: An exploratory analysis of CADTH common drug review.* Research Involvement and Engagement, 2016. **2**(1).
3. Gargon, E., S.L. Gorst, and P.R. Williamson, *Choosing important health outcomes for comparative effectiveness research: 5th annual update to a systematic review of core outcome sets for research.* PLoS ONE, 2019. **14**(12): p. 1-18.
4. Keeney, S., F. Hasson, and H. McKenna, *The Delphi Technique in Nursing and Health Research*. 2010: Wiley, Blackwell.
5. Gargon, E., et al., *Choosing important health outcomes for comparative effectiveness research: 6th annual update to a systematic review of core outcome sets for research.* PLoS ONE, 2021. **16**(1): p. 1-12.
6. Gargon, E., P.R. Williamson, and B. Young, *Improving core outcome set development: qualitative interviews with developers provided pointers to inform guidance.* Journal of Clinical Epidemiology, 2017. **86**: p. 140-152.
7. Biggane, A.M., et al., *Participating in core outcome set development via Delphi surveys: qualitative interviews provide pointers to inform guidance.* BMJ open, 2019. **9**(11): p. e032338.
8. Young, B. and H. Bagley, *Including patients in core outcome set development: issues to consider based on three workshops with around 100 international delegates.* Research Involvement and Engagement, 2016. **2**(1).
9. Gargon, E., et al., *Choosing Important Health Outcomes for Comparative Effectiveness Research: A Systematic Review.* PLoS ONE, 2014. **9**(6): p. 1-12.
10. Gorst, S.L., et al., *Choosing Important Health Outcomes for Comparative Effectiveness Research: An Updated Review and User Survey.* PLoS ONE, 2016. **11**(1): p. 1-12.
11. Gorst, S.L., et al., *Choosing important health outcomes for comparative effectiveness research: An updated review and identification of gaps.* PLoS ONE, 2016. **11**(12).
12. Davis, K., et al., *Choosing important health outcomes for comparative effectiveness research: An updated systematic review and involvement of low and middle income countries.* PLoS ONE, 2018. **13**(2): p. 1-14.

For peer review only - http://bmjopen.bmj.com/site/about/guidelines.xhtml

13. Gargon, E., et al., *Choosing important health outcomes for comparative effectiveness research: 4th annual update to a systematic review of core outcome sets for research.* PLoS ONE, 2018. **13**(12): p. 1-15.
14. Deborah, A.H., et al., *The COMiT'ID Study: Developing Core Outcome Domains Sets for Clinical Trials of Sound-, Psychology-, and Pharmacology-Based Interventions for Chronic Subjective Tinnitus in Adults.* Trends in Hearing, 2018. **22**.
15. Potter, S., et al., *Development of a core outcome set for research and audit studies in reconstructive breast surgery.* British Journal of Surgery, 2015. **102**(11): p. 1360.
16. Williamson, P.R., et al., *Controversy and Debate Series on Core Outcome Sets. Paper 4: Debate on Paper 1 from the perspective of COMET [Core Outcome Measures in Effectiveness Trials]*. 2020. p. 222-224.
17. Brookes, S.T., et al., *Three nested randomized controlled trials of peer-only or multiple stakeholder group feedback within Delphi surveys during core outcome and information set development.* Trials, 2016. **17**(1).
18. Fish, R., et al., *"Vicarious thinking" was a key driver of score change in Delphi surveys for COS development and is facilitated by feedback of results.* Journal of Clinical Epidemiology, 2020. **128**: p. 118-129.
19. MacLennan, S., et al., *A randomized trial comparing three Delphi feedback strategies found no evidence of a difference in a setting with high initial agreement.* Journal of Clinical Epidemiology, 2018. **93**: p. 1-8.
20. Blackwood, B., et al., *Core Outcomes in Ventilation Trials (COVenT): protocol for a core outcome set using a Delphi survey with a nested randomised trial and observational cohort study*. 2015, Springer Nature.
21. Fish, R., *Development of a core outcome set for trials of chemoradiotherapy for anal squamous cell carcinoma*. 2018, University of Manchester.
22. Jamie, J.K., et al., *Core Outcome Set-STAndards for Development: The COS-STAD recommendations.* PLoS Medicine, 2017. **14**(11): p. e1002447-e1002447.
23. Miles, D.W., et al., *Developing a roadmap to improve trial delivery for under-served groups: results from a UK multi-stakeholder process.* Trials, 2020. **21**(1): p. 1-9.
24. Harriet, S., et al., *Defining and evaluating novel procedures for involving patients in Core Outcome Set research: creating a meaningful long list of candidate outcome domains.* Research Involvement and Engagement, 2018. **4**(1): p. 1-12.
25. Crudgington, H., et al. *Core Health Outcomes in Childhood Epilepsy (CHOICE): Development of a core outcome set using systematic review methods and a Delphi survey consensus*. 2019. Netherlands: John Wiley & Sons Ltd.
26. Staniszewska, S., et al., *GRIPP2 reporting checklists: tools to improve reporting of patient and public involvement in research.* Research Involvement and Engagement, 2017. **3**(1): p. 1-11.
27. Jamie, J.K., et al., *Core Outcome Set-STAndards for Reporting: The COS-STAR Statement.* PLoS Medicine, 2016. **13**(10): p. e1002148-e1002148.

Footnotes

Twitter @COMETinitiative

Author statement

Contributors: Conceptualization: HB, PRW, BY; Funding acquisition: PRW; Investigation: HB, PRW, BY; Methodology: HB, PRW, BY; Writing – original draft: HB; Writing – review & editing: HB, PRW, BY

For peer review only - http://bmjopen.bmj.com/site/about/guidelines.xhtml

Funding: HB is supported by the National Institutes for Health Research (NIHR) through award number NF-SI_0513-10025. The views expressed in this article are those of the author(s) and not necessarily those of the NIHR, or the Department of Health and Social Care. PW is also supported by the Medical Research Council (MRC) Trials Methodology Research Partnership (grant reference MR/S014357/1).

Conflicts of interests: PW & HB are members of the COMET Management Group, BY and HB are members of the COMET PoPPIE Working Group.

Ethics approval: This systematic review was based on published data. Therefore, obtaining ethical approval was unnecessary.

Patient consent for publication: Not applicable

Data availability statement: On reasonable request from the first author

For peer review only

Tables

Table 1 – Scope of the Core Outcome Set

Core Outcome Set Scope	
**Health area**	**n (%)**
Anaesthesia & pain control	1 (1%)
Blood disorders	1 (1%)
Cancer	9 (12%)
Cancer/ Child health	1 (1%)
Child health	1 (1%)
Child health/ Ear, nose & throat	1 (1%)
Child health/ Gastroenterology	1 (1%)
Ear, nose & throat	4 (5%)[a]
Endocrine & metabolic	3 (4%)
Eyes & Vision	1 (1%)
Gastroenterology	6 (8%)
Healthcare of older people	2 (3%)
Heart & circulation	3 (4%)
Heart & Circulation and skin	3 (4%)[a]
Kidney disease	2 (3%)
Lungs & airways	2 (3%)
Mental health	1 (1%)
Neonatal care	1 (1%)
Neurology	4 (5%)[b]
Neurology / eyes & vision	1 (1%)
Orthopaedics & trauma	6 (8%)
Other	2 (3%)
Overweight / obesity	1 (1%)
Pregnancy & childbirth	11 (14%)[c]
Rehabilitation	1 (1%)
Rehabilitation, Rheumatology	1 (1%)

Rheumatology	2 (3%)
Skin	4 (5%)
Tobacco, drugs & alcohol dependence	1 (1%)
**Adults / Children**	**n (%)**
Adults	45 (58%)
Both adults and children	18 (23%)
Children	11 (14%)
Not reported	4 (5%)
**Gender**	**n (%)**
Male only	2 (3%)
Female only	8 (10%)
Both	68 (87%)
**Intervention**	**n (%)**
Any	49 (63%)
Drug	4 (5%)
Psychological	3 (4%)
Surgery	7 (9%)
Other[d]	15 (19%)
**Countries (all participants)**	**n (%)**
1 only	11 (19%)
2-10	14 (24%)
11-10	8 (14%)
20-30	13 (22%)
>30	13 (22%)
Not reported / unclear	19

Footnotes:

[a] Includes articles reporting 3 COS studies

[b]Includes articles reporting 2 COS studies

[c] Includes 2 articles reporting 2 COS studies

[d]Other: active surveillance anaesthetic techniques; behavioural; chemoradiotherapy; ECMO; gene therapy; haemodialysis; health care transition; interdisciplinary multimodal pain therapy; medication review; physical activity intervention; pre-pregnancy care; procedure (induction of labour); rehabilitation; sound-based interventions; visual screening / assessment.

For peer review only

Table 2 - Study characteristics of the Delphi studies

**Study characteristics**	
**Methods to explore patients' views on important outcomes prior to the Delphi study[a]**	**n (%)**
Patient interviews	20 (26%)
Survey	12 (12%)[b]
Nominal group technique	3(4%)
Focus groups	4 (5%)
Not reported / unclear	47
**Pilot Delphi undertaken**	**n (%)**

For peer review only - http://bmjopen.bmj.com/site/about/guidelines.xhtml

Pilot study reported	28 (36%)[c]
**Patient and Public Involvement (PPI)**	**n (%)**
PPI reported	31 (40%)
**Method of delivery (LE)**	**n (%)**
Electronic	39 (74%)
Post	4 (8%)
Face to face	3 (6%)
Mixture of approaches	7 (13%)
Not reported	19
Unclear	6
**Reminders**	**n (%)**
1 reminder between rounds	10 (31%)
More than one reminder between rounds	22 (69%)
Reminders sent but number of reminders not reported	12
Not reported	46[d]
**Incentives (patient participants)**	**n (%)**
Yes (monetary incentive / voucher)	3 (38%)
Yes (non-monetary incentive)[e]	3 (38%)
Incentive not offered	2 (25%)
Not reported	70
**Language used with patients**	**n (%)**
Translation	10 (20%)
Conducted in English (specifically stated)	19 (37%)
Native language (implicit)	22 (43%)
Not reported	27
**Participant recruitment source & approach[f]**	
Recruitment source (patients)	n (%)
Patient organisation	43 (62%)
Clinic / Treatment centre	31 (45%)
Social media	19 (28%)
PPI group (external to the COS study)	14 (20%)
Contacts of Steering Committee / Management Group	7 (10%)
Snowball sampling	10 (15%)
Research database	6 (9%)
Other[g]	See footnote
Unclear[h]	3
Not reported	6
**Recruitment approach (patients)**	**n (%)**
Email invitation	42 (74%)
Postal invitation	5 (9%)
Telephone invitation	4 (7%)
Information provided in clinic	7 (12%)
Poster / newsletter	7 (12%)
e-source (website / social media)	15 (30%)
Recruitment approach unclear	5
Not reported	16
**Participant characteristics reported**	

For peer review only

For peer review only - http://bmjopen.bmj.com/site/about/guidelines.xhtml

Patient participants	n (%)
Age	39 (50%)[i]
Gender	44 (56%)[j]
Socio-economic / education	8 (10%)[k]
Ethnicity	8 (10%)[l]
Marital status	7 (9%)
Experience of condition	24 (31%)
Experience of treatment	15 (19%)
Other[m]	See footnote

Footnotes:

[a]Some studies used more than one approach to explore patients views on outcomes prior to the Delphi.

[b]Including 6 studies in which patients identified outcomes in what the authors referred to as 'round 1'.

[c]Including 3 studies where pilots were without patients

[d]Including 12studies where reminders were sent but the number of reminders was not reported

[e]All non-monetary were certificates and reported in a single article

[f]More than one recruitment source / approach may have been used.

[g]Other included through a professional organisation (n=2), a conference attended by patients (n=3, 3 COS from the same article), previous participation in a research study (n=4) and participating researchers identified patients (n=1)

[h]Additional articles partially unclear, recruitment source (n=3), recruitment approach (n=3)

[i]Including 5 studies where age was reported collectively for both patients and professionals and 1 study where age reported for parent's child only

[j]Including 12 where COS study was specifically targeted at one gender and 9 studies where gender was reported collectively for both patients and professionals.

[k]Including 1 study where education was reported collectively for both patients and professionals

[l]Including 2 studies where ethnicity was reported collectively for both patients and professionals.

[m]Other- previous participation in research (n=2, both of which reported collectively for both patients and professionals), number of children (n=1), home type (n = 1)

Table 3 – Delphi specific survey issues

Duration of rounds				
Round duration	n (%)			
Time for each round	< 2 weeks	2– 4 weeks	>4 weeks	Not reported / not clear / n/a
Round 1	1 (3%)	23 (70%)	9 (27%)	45

For peer review only - http://bmjopen.bmj.com/site/about/guidelines.xhtml

Round 2	1 (3%)	25 (78%)	6(19%)	46
Round 3	0	16 (80%)	4 (20%)	58
**Scoring Systems and Feedback Approaches**				
**Scoring system (Round 1)**				**n (%)**
1-9 / 1-10[a]				52 (70%)[b]
0-4/1-4 / 1-5				12 (16%)
9/10/12 most important outcomes				4 (5%)
Yes/no/don't know or agree/disagree/unsure				7 (9%)
Not reported				2
Unclear				1
**Source of stakeholder feedback Round 2**				**n (%)**
All stakeholder groups combined				10[c] (21%)
Stakeholder groups reported separately				21 (44%)
Own Stakeholder group only				10[d] (21%)
Stakeholder groups reported separately and all stakeholder groups combined				5 (10%)
SWAT[e] – different groups saw different feedback				1 (4%)
N/a patients only took part in 1 round				13
Not reported				13
Unclear				4
**Feedback type reported[f]**				**n (%)**
Graphical feedback[g]				17 (40%)
Numerical frequencies				24 (56%)
Summary statistics[g]				15 (35%)[h]
Dispersion / distribution of scores				28 (65%)
Anonymised comments from prior round				2 (5%)
N/a patients only voted in one round				13
Not reported				22

Footnotes

[a]Only two studies used 1-10

[b]Children in one of these studies used 1-3 scale and Caregivers in another study scored differently to patients in one of these studies – patients used score cards

[c]Including one study which also provided the patient group scores and one study in which participants could request feedback by stakeholder group

[d]Including one study which also provided combined scores for all

[e]SWAT – Study Within a Trial

[f]Studies could report more than one type of feedback

[g]Excludes anywhere it was unclear whether the feedback type was reported

[h]10 studies reported only summary statistics

Table 4 – Response rates

Round	Participation[a]	Median, Min, Max
1	Patients invited and completed (n=20)	59%, 11%, 95%

For peer review only - http://bmjopen.bmj.com/site/about/guidelines.xhtml

	Professionals invited and completed (n = 20)	52%, 19%, 93%
	Ratio of Professionals to patients (n=62)	2.7, 4.1, 0.4, 23
2	Patients invited and completed (n=44)	84%, 32%, 100%
	Professionals invited and completed (n=46)	85%, 43%, 100%
3	Patients invited and completed (n=20)	91%, 50%, 100%
	Professionals invited and completed (n=24)	91%, 78%, 100%

[a] In round 2 and / or round 3 some studies described non-responders to a previous round being invited into the round (this could be both patient and professional previous responders or just one type of previous responder). These studies were excluded from analysis of round 2 and / or round 3 response rate data for the relevant category of respondent. Round 1 participation rates were available for studies where the denominator was known (i.e. the number of people invited).

Table 5 – Association between patient response rate and PPI, piloting and recruitment source

Factor	Round[a]	Factor category	Patients- median response rate, min, max
**PPI**	**1**	PPI (n=6)	62%, 36%, 77%
		PPI not reported (n=14)	59%, 11%, 95%
	**2**	PPI (n=22)	78%, 32%, 94%
		PPI not reported (n = 22)	86%, 50%, 100%
	**3**	PPI (n=9)	92%, 71%, 100%
		PPI not reported (n =11)	90%, 50%, 100%
**Piloting**	1	Piloting (n = 10)	61%, 36%, 95%
		No piloting reported (n=10)	58%, 11%, 91%
	2	Piloting (n =21)	84%, 41%, 100%
		No piloting reported (n = 23)	83%, 32%, 100%
	3	Piloting (n =9)	92%, 71%, 100%
		No piloting reported (n=11)	89%, 50%, 100%
**Recruitment source**	2	Treatment Centre (n=6)	89%, 83%, 90%
		Patient organisation (n = 20)	77%, 32%, 100%
		Treatment centre and patient organisation (n = 11)	77%, 50%, 93%
		Neither treatment centre nor patient organisation (n = 5)	94%, 90%, 100%

For peer review only

For peer review only - http://bmjopen.bmj.com/site/about/guidelines.xhtml

		Nothing reported on recruitment source (n = 2)	92%, 84%, 100%
**Reminders**	2	Reminders (n = 30)	82, 32,96
		No reminders reported (n = 14)	86, 57, 100

Footnote

[a]19 studies with round 1 data on participation rate, 44 studies with round 2 completion rate and 20 with round 3 completion rate data.

For peer review only

For peer review only - http://bmjopen.bmj.com/site/about/guidelines.xhtml

Patient participation in Delphi surveys to develop core outcome sets: systematic review

Authors: Barrington H.J.[1], Young B.[1] & Williamson P.R.[1]

Author affiliations: [1]University of Liverpool, Liverpool, U.K.

Supplementary tables

Table 2a - Study development and design characteristics of the Delphi studies

Study design & development characteristics	
**Number of rounds where patients participated**	**n (%)**
1	13 (17%)
2	28 (36%)
3	37 (47%)
**Number of stakeholder participant categories**	**n (%)**
2	31 (40%)
3	20 (26%)
4	16 (21%)
5	10 (13%)
6	1 (1%)
**Number of reported items per round**	**Descriptive statistics[a]**
Round 1 (n=71)	Median = 46, Min = 9, Max = 130
Round 2 (n=53)	Median = 49, Min = 8, Max = 130
Round 3 (n=28)	Median = 37, Min = 7, Max = 114

Footnote

[a]excluding not reported, n/a, unclear

Table 2b – Study characteristics (professional participants)

Professional recruitment source & approach[a]	
**Professional recruitment source**	**n (%)**
Professional organisation	49 (70%)
Publication authors (including Cochrane authors)	22 (31%)
Research study	13(19%)
Research group / consortium /CTU groups (including Cochrane group)	32 (46%)
Steering group members / contacts / University contacts	14 (20%)
Treatment centres	15 (21%)
Snowball sampling	25 (36%)
Other[b]	See below
Not reported	8

For peer review only - http://bmjopen.bmj.com/site/about/guidelines.xhtml

Professional recruitment approach	n (%)
Email invitation	50 (91%)
Postal invitation	4 (7%)
Handed invitation	4 (7%)
Newsletter / webpage	5 (9%)
Unclear	3
Not reported	20
**Participant characteristics reported**	
**Professional participants**	**n (%)**
Clinical experience	20 (26%)
Research experience	9 (12%)[c]
Gender	24 (31%)[d]
Age	21 (27%)e
Ethnicity	4 (5%)c
Education	3 (4%)f

Footnotes

[a] More than one recruitment source could be used

[b] Other included journal editorial groups (9), through informal mailing lists (n=2), members of steering committee (n=2), conference / conference special interest group (n=4) email discussion group / special interest group (n=4), research funding organisation (n = 2), audit participant (n=1)

[c]Includes 2 studies where characteristic reported collectively on research experience and ethnicity for PE and LE

[d]Includes 9 studies where characteristic reported collectively on gender for professionals and patients

[e]Includes 5 studies where characteristic reported collectively for professionals and patients

[f]Includes 1 study where characteristic reported collectively for professionals and patients

Table 3a – Delphi characteristics rounds 2 and 3

Scoring System Rounds 2 &3		
**Scoring system**	**Round 2 n (%)**	**Round 3 n (%)**
1-9 / 1-10[a]	52 (85%)	26 (77%)
0-4/1-4 / 1-5	4 (7%)	3 (9%)
9/10/12 most important outcomes	2 (3%)[b]	1 (3%)
Yes/no/don't know or agree/disagree/unsure	2 (3%)	1 (3%)
Yes/no/include in COS & Essential and recommended outcomes	n/a	3 (9%)
Domain inner core, middle ring, outer ring	1 (2%)	n/a
Not reported	2	1
Unclear	2	2
n/a patients only in 1 round	13	13
n/a only 2 rounds	0	28
**Feedback**		
**Feedback type Round 3**		**n (%)**

For peer review only - http://bmjopen.bmj.com/site/about/guidelines.xhtml

All stakeholder groups combined	7 (28%)
Stakeholder groups reported separately	9 (36%)
Own stakeholder group	1 (4%)
Each stakeholder group & all stakeholder groups combined	3 (12%)
Own stakeholder group & all stakeholder groups combined	3 (12%)
SWAT	2 (8%)
Not reported	6
N/a only 2 rounds	28
N/a patients only took part in one round	13
Unclear	6

Footnotes

[a]Only two studies used 1-10

[b]Caregivers scored differently to patients in one of these studies – patients used score cards

For peer review only - http://bmjopen.bmj.com/site/about/guidelines.xhtml

Supplementary Figure 1 - Preferred Reporting Items for Systematic Reviews and Meta-Analyses flowchart of identification of eligible studies from the COMET database. Data were extracted from the COS systematic reviews

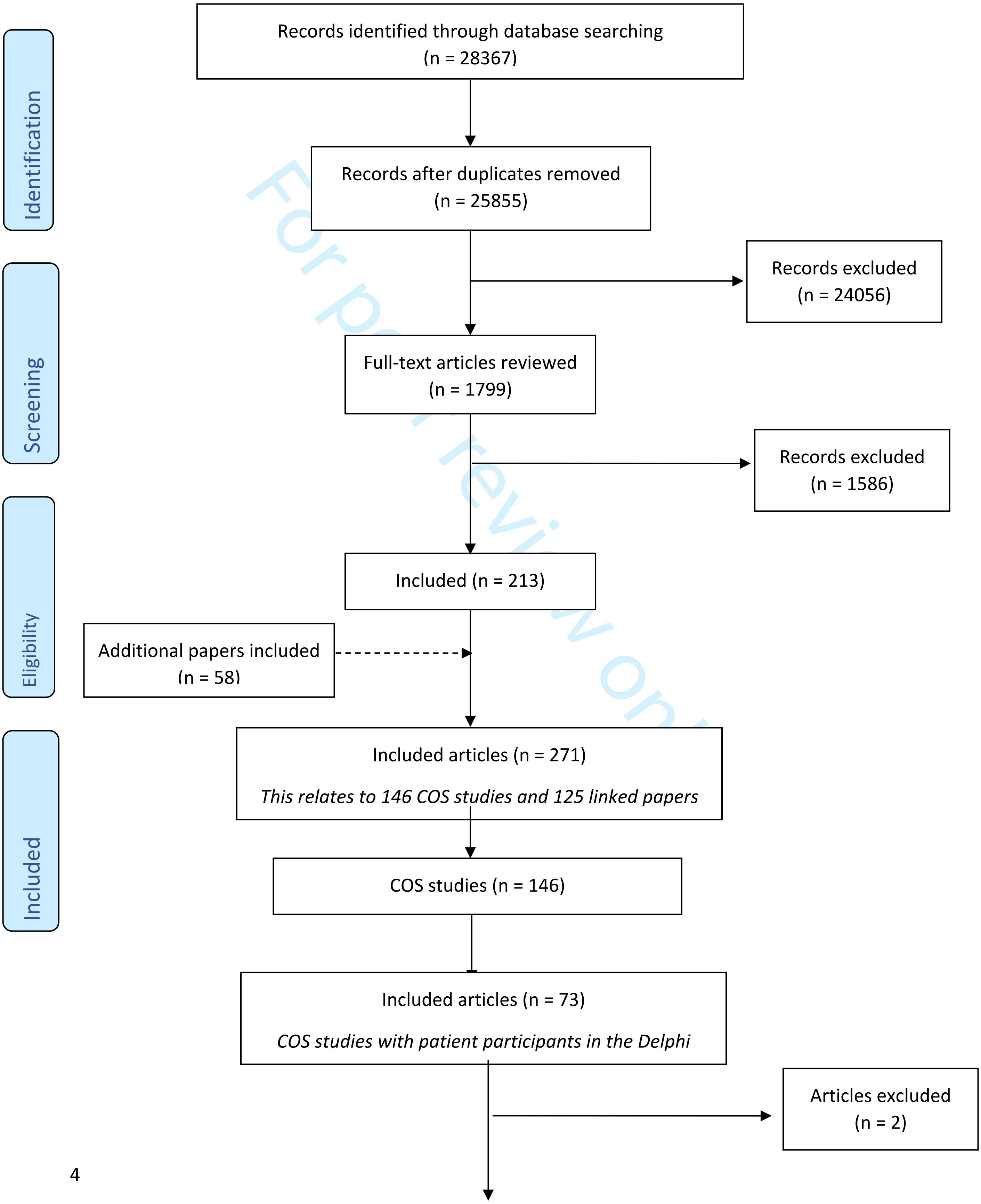

For peer review only - http://bmjopen.bmj.com/site/about/guidelines.xhtml

Included articles (n = 71)

COS studies with more than one patient participant in the Delphi

For peer review only

For peer review only - http://bmjopen.bmj.com/site/about/guidelines.xhtml

Reporting checklist for systematic review and meta-analysis.

Based on the PRISMA guidelines.

Instructions to authors

Complete this checklist by entering the page numbers from your manuscript where readers will find each of the items listed below.

Your article may not currently address all the items on the checklist. Please modify your text to include the missing information. If you are certain that an item does not apply, please write "n/a" and provide a short explanation.

Upload your completed checklist as an extra file when you submit to a journal.

In your methods section, say that you used the PRISMAreporting guidelines, and cite them as:

Moher D, Liberati A, Tetzlaff J, Altman DG, The PRISMA Group. Preferred Reporting Items for Systematic Reviews and Meta-Analyses: The PRISMA Statement

		Reporting Item	Page Number
**Title**			
	#1	Identify the report as a systematic review, meta-analysis, or both.	1
**Abstract**			

For peer review only

Structured summary	#2	Provide a structured summary including, as applicable: background; objectives; data sources; study eligibility criteria, participants, and interventions; study appraisal and synthesis methods; results; limitations; conclusions and implications of key findings; systematic review registration number	1
**Introduction**			
Rationale	#3	Describe the rationale for the review in the context of what is already known.	2
Objectives	#4	Provide an explicit statement of questions being addressed with reference to participants, interventions, comparisons, outcomes, and study design (PICOS).	2
**Methods**			
Protocol and registration	#5	Indicate if a review protocol exists, if and where it can be accessed (e.g., Web address) and, if available, provide registration information including the registration number.	3
Eligibility criteria	#6	Specify study characteristics (e.g., PICOS, length of follow-up) and report characteristics (e.g., years considered, language, publication status) used as criteria for eligibility, giving rational	3
Information sources	#7	Describe all information sources in the search (e.g., databases with dates of coverage, contact with study authors to identify additional studies) and date last searched.	3

For peer review only

Search	#8	Present full electronic search strategy for at least one database, including any limits used, such that it could be repeated.	3
Study selection	#9	State the process for selecting studies (i.e., for screening, for determining eligibility, for inclusion in the systematic review, and, if applicable, for inclusion in the meta-analysis).	3
Data collection process	#10	Describe the method of data extraction from reports (e.g., piloted forms, independently by two reviewers) and any processes for obtaining and confirming data from investigators.	3
Data items	#11	List and define all variables for which data were sought (e.g., PICOS, funding sources), and any assumptions and simplifications made.	3
Risk of bias in individual studies	#12	Describe methods used for assessing risk of bias in individual studies (including specification of whether this was done at the study or outcome level, or both), and how this information is to be used in any data synthesis.	N/A
Summary measures	#13	State the principal summary measures (e.g., risk ratio, difference in means).	3/4
Planned methods of analyis	#14	Describe the methods of handling data and combining results of studies, if done, including measures of consistency (e.g., I2) for each meta-analysis.	3/4

For peer review only

Risk of bias across studies	#15	Specify any assessment of risk of bias that may affect the cumulative evidence (e.g., publication bias, selective reporting within studies).	N/A
Additional analyses	#16	Describe methods of additional analyses (e.g., sensitivity or subgroup analyses, meta-regression), if done, indicating which were pre-specified.	N/A
**Results**			
Study selection	#17	Give numbers of studies screened, assessed for eligibility, and included in the review, with reasons for exclusions at each stage, ideally with a flow diagram.	4
Study characteristics	#18	For each study, present characteristics for which data were extracted (e.g., study size, PICOS, follow-up period) and provide the citation.	4
Risk of bias within studies	#19	Present data on risk of bias of each study and, if available, any outcome-level assessment (see Item 12).	N/A
Results of individual studies	#20	For all outcomes considered (benefits and harms), present, for each study: (a) simple summary data for each intervention group and (b) effect estimates and confidence intervals, ideally with a forest plot.	N/A
Synthesis of results	#21	Present the main results of the review. If meta-analyses are done, include for each, confidence intervals and measures of consistency.	4

For peer review only

Risk of bias across studies	#22	Present results of any assessment of risk of bias across studies (see Item 15).	N/A
Additional analysis	#23	Give results of additional analyses, if done (e.g., sensitivity or subgroup analyses, meta-regression [see Item 16]).	N/A
**Discussion**			
Summary of Evidence	#24	Summarize the main findings, including the strength of evidence for each main outcome; consider their relevance to key groups (e.g., health care providers, users, and policy makers	6-8
Limitations	#25	Discuss limitations at study and outcome level (e.g., risk of bias), and at review level (e.g., incomplete retrieval of identified research, reporting bias).	6-8
Conclusions	#26	Provide a general interpretation of the results in the context of other evidence, and implications for future research.	8
**Funding**			
Funding	#27	Describe sources of funding or other support (e.g., supply of data) for the systematic review; role of funders for the systematic review.	9

None The PRISMA checklist is distributed under the terms of the Creative Commons Attribution License CC-BY. This checklist can be completed online using https://www.goodreports.org/, a tool made by the EQUATOR Network in collaboration with Penelope.ai

For peer review only

BMJ Open

Patient participation in Delphi surveys to develop core outcome sets: systematic review

Journal:	*BMJ Open*
Manuscript ID	bmjopen-2021-051066.R1
Article Type:	Original research
Date Submitted by the Author:	14-Jul-2021
Complete List of Authors:	Barrington, Heather; University of Liverpool, Health Data Science<br>Young, Bridget; University of Liverpool, Department of Psychological Sciences<br>Williamson, Paula; The University of Liverpool, Biostatistics
**Primary Subject Heading**:	Research methods
Secondary Subject Heading:	Health services research
Keywords:	STATISTICS & RESEARCH METHODS, GENERAL MEDICINE (see Internal Medicine), SURGERY

SCHOLARONE™
Manuscripts

I, the Submitting Author has the right to grant and does grant on behalf of all authors of the Work (as defined in the below author licence), an exclusive licence and/or a non-exclusive licence for contributions from authors who are: i) UK Crown employees; ii) where BMJ has agreed a CC-BY licence shall apply, and/or iii) in accordance with the terms applicable for US Federal Government officers or employees acting as part of their official duties; on a worldwide, perpetual, irrevocable, royalty-free basis to BMJ Publishing Group Ltd ("BMJ") its licensees and where the relevant Journal is co-owned by BMJ to the co-owners of the Journal, to publish the Work in this journal and any other BMJ products and to exploit all rights, as set out in our licence.

The Submitting Author accepts and understands that any supply made under these terms is made by BMJ to the Submitting Author unless you are acting as an employee on behalf of your employer or a postgraduate student of an affiliated institution which is paying any applicable article publishing charge ("APC") for Open Access articles. Where the Submitting Author wishes to make the Work available on an Open Access basis (and intends to pay the relevant APC), the terms of reuse of such Open Access shall be governed by a Creative Commons licence – details of these licences and which Creative Commons licence will apply to this Work are set out in our licence referred to above.

Other than as permitted in any relevant BMJ Author's Self Archiving Policies, I confirm this Work has not been accepted for publication elsewhere, is not being considered for publication elsewhere and does not duplicate material already published. I confirm all authors consent to publication of this Work and authorise the granting of this licence.

For peer review only

Patient participation in Delphi surveys to develop core outcome sets: systematic review

Authors: Barrington H.J.[1], Young B.[1] & Williamson P.R.[1]

Author affiliations: [1]University of Liverpool, Liverpool, U.K.

Author email addresses: heather.barrington@liverpool.ac.uk, byoung@liverpool.ac.uk, prw@liverpool.ac.uk.

Correspondence to: Heather Barrington: heather.barrington@liverpool.ac.uk

Keywords: core outcome set; systematic review; patient participation; Delphi surveys

Word count: 3432

For peer review only

Abstract

Objectives: To describe the design and conduct of core outcome set (COS) studies that have included patients as participants, exploring how study characteristics might impact their response rates.

Design: Systematic review of COS studies published between 2015 and 2019 that included more than one patient, carer or representative as participants (hereafter referred to as patients for brevity) in scoring outcomes in a Delphi.

Results: There were variations in the design and conduct of COS studies that included patients in the Delphi process, including differing: scoring and feedback systems, approaches to recruiting patients, length of time between rounds, use of reminders, incentives, patient and public involvement and piloting. Minimal reporting of participant characteristics and a lack of translation of Delphi surveys into local languages were found. Additionally, there were indications that studies which recruited patients through treatment centres had higher round 2 response rates than studies recruiting through patient organisations.

Conclusions: Variability was striking in how COS Delphi surveys were designed and conducted to include patient participants and other stakeholders. Future research is needed to explore what motivates patients to take part in COS studies and what factors influence COS developer recruitment strategies. Improved reporting would increase knowledge of how methods affect patient participation in COS Delphi studies.

Article summary

Strengths and limitations of this study

- This is the first systematic review of patient participation in Delphi surveys for core outcome set development.

For peer review only - http://bmjopen.bmj.com/site/about/guidelines.xhtml

- This comprehensive review explored both study characteristics and recruitment and retention rates amongst patients.
- The findings are limited by reporting issues in the reviewed studies, especially on recruitment and few studies reported how many individuals received the initial invitation to participate.
- Other reporting issues, including on patient and public involvement, limit the conclusions that can be drawn from this review.

Background

Patients and health care professionals need evidence about what treatments work best to inform their health care decisions. The results of clinical trials are, however, often difficult to compare due to a lack of standardisation in the outcomes measured for the same health condition and challenges with reporting bias[1]. In addition, including the perspectives of patients on what outcomes matter to them is crucial[2] Core outcome sets (COS) are a potential solution to these problems, providing standardised sets of outcomes, developed and agreed upon by key stakeholders, including patients.

COS are developed through iterative consensus building processes. Commonly a systematic review and sometimes qualitative interviews with patients are used to explore patients' views on outcomes and generate a long list of potential outcomes. These outcomes are then taken forward into a consensus process, most gathering views through a Delphi survey and ratifying these results at a consensus meeting to agree upon a COS[3]. Delphi participants are invited to score outcomes in several survey 'rounds', considering the feedback of other expert groups as part of the process. Delphi surveys lend themselves to e-surveys and as such can be widely distributed, however, like other questionnaires, these surveys are prone to low response rates[4].

Patient participation in COS studies has increased over recent years, with Gargon et al[5] reporting 77% of published COS studies included patients or their representatives (for example, carers or patient advocates). While this paper focusses largely on patient participation in COS, it is important to distinguish between this and patient involvement in COS studies. When patients participate, they are contributing data on which outcomes to prioritise, for example scoring outcomes in Delphi studies. When patients are involved in COS studies they are helping to design and oversee the COS study from a patient / public perspective. There are several challenges in including patient participants in COS and indeed there are indications some COS developers 'problematise' patient participation[6], highlighting for example, the tendency for patients to rate many outcomes highly. Biggane et al[7] found that patients without prior experience of Delphi surveys expressed difficulty understanding both the purpose of the COS and particular aspects of the surveys. Young and Bagley[8] called for further research exploring how patient input is currently being sought in COS studies and to understand more about the challenges of including and engaging patients in COS development.

To the authors' knowledge no review of patient participation in COS Delphi studies has previously been published. We have undertaken a systematic review of recent COS studies that have included patients in their COS Delphi, to describe how these studies have been designed and conducted and whether participation rates were linked with the study design variables: recruitment source, PPI and reminders. By identifying challenges in recruiting and retaining patients in COS studies this review aimed to inform strategies to optimise the participation of patients in future COS studies.

For peer review only - http://bmjopen.bmj.com/site/about/guidelines.xhtml

Methods

The protocol is available at: www.comet-initiative.org/Studies/Details/1824

Study selection

Inclusion criteria Eligible COS studies were those identifying outcomes for use in research, published between 1st January 2015 and 31st December 2019 (to reflect current practice), and including more than one patient, carer or their representative as a participant (hereafter referred to as patients for brevity) in scoring outcomes in a Delphi as part of the process.

Identification of relevant studies: Studies were identified through the COMET Initiative database. How studies are identified for inclusion in this database has previously been described[3, 5, 9-13]. Briefly, eligible studies for the database were those that employed methodology to gain consensus as to which outcome domains or outcomes should be measured in clinical trials or other forms of health research. Any studies that described the update of an existing COS are included in the database as linked papers to the original COS. Eligible studies are added to the database as they are identified, and an annual systematic review of these is published to ensure the database is kept current**.**

Studies meeting the criteria for our review were selected from the aforementioned database, linked studies were not included. Where authors referred the reader to the protocol in the methods section of their article, these protocols were also reviewed. Studies reporting updates to COS studies that were already in the COMET database were not included in the current review.

Data extraction

A data extraction template was developed including the following domains:

- **Study scope** – Health area; the population; intervention type; location (participating countries).
- **Study development and design** – Methods to explore patients' views on outcomes; survey language and translation, participant groups represented; number of rounds; number of outcomes in each round; reported PPI and piloting; scoring and feedback systems used; use of reminders and other incentives; recruitment sources and methods.
- **Study conduct and results** – Reporting of participant characteristics; response rates in each round by participant group; ratio professionals (PE, i.e. participants not providing a patient perspective, such as health care professionals and researchers): patients in round 1.

Some studies had included patients and other stakeholders earlier in their COS, for example in generating a list of outcomes, and authors sometimes referred to these as 'rounds'. Only rounds relating to the scoring of outcomes were included in this review. Data extraction was undertaken by one person (HB) with checking of certain technical aspects, such as the methods of feedback, by a second person (PW).

Data analysis

In addition to describing how studies had been designed and conducted, we were keen to explore whether participation rates were linked with other study design variables. We anticipated, for example, that more personalised recruitment approaches or the use of incentives or reminders

For peer review only - http://bmjopen.bmj.com/site/about/guidelines.xhtml

might impact response rates and that steps to enhance the design of surveys such as patient and public involvement (PPI) and piloting might also impact patient participant responses. Additionally, we wished to explore whether the recruitment source used in a study influenced patient participation. The two most commonly used recruitment sources were patient organisations and treatment centres, therefore these were chosen for comparison. As several studies used both these sources we also explored their combined influence on participation.

Patient and public involvement

Patients and the public were not involved in the design, conduct, or reporting of this review of previously published data.

Results

The PRISMA diagram for the review is presented in Supplementary Figure 1. From a total of 146 COS studies published between 2015 and 2019, 73 COS studies were initially identified as eligible, however 2 of these were subsequently excluded as only one patient had participated. Of the 71 included COS studies, 66 reported on a single core outcome set. The remaining 5 studies reported on a total of 12 COS. For example, one article by Hall et al[14] reported on three COS for three different interventions in tinnitus. Patients could complete any or all of these Delphi surveys so recruitment and retention data for each of these COS studies could be different. After discussion it was decided to treat each COS individually. Of the five articles which reported on more than one COS, two each reported on three COS, and three articles each reported on two COS. In total, therefore 78 COS studies are included in this review. In thirteen of the COS studies, patients participated in only one round of scoring in the Delphi.

Study scope

Table 1 illustrates the scope of the included studies. The COS studies represented a broad range of health areas, with pregnancy and childbirth (14%, n =11) and cancer (12%, n = 9) being the most common. Whilst the COS were predominantly developed for adults (58%, n =45), 14% (n=11) were for children. Most COS were developed for any intervention (63%, n = 49). The median number of countries participating in the COS studies was 16 (in 18 studies the number of countries was either not reported or unclear), maximum 73, and 13% (n=10) were conducted in a single country. Where data was given for numbers of countries from which the patient participants were drawn, the maximum number of countries was 21.

Table 1 around here

Study characteristics

The variation in study characteristics can be seen in Table 2. In preparation for the Delphi study, the most common method used to explore patients' views on outcomes was by interview (n = 20, 26%).

Thirty six percent of studies (n = 28) described piloting the Delphi, whilst patient involvement in the study design or delivery provided in the main COS report was reported by 40% of studies (n=31), although the detail around the PPI and piloting was generally minimal.

Most COS studies were delivered electronically to patients (74%, n =39) and 59% (n=23) of these were delivered using the DelphiManager software developed by the COMET Initiative. Of the 51 studies that either reported on language used or where it was implicit in the description, 20% (n=10) of studies described offering some form of translation of the study materials (including 3 COS studies

For peer review only - http://bmjopen.bmj.com/site/about/guidelines.xhtml

For peer review only

in one article). Just over half the studies reported using reminders (56%, n=44). Only 8% (n=6) of studies described using incentives, 3 monetary incentives and 3 non-monetary (3 COS from the same article).

A range of recruitment sources were used to recruit patients and some studies used multiple sources. Patient organisations (62%, n = 43) and treatment centres (45%, n = 31) were the most common. The most common method of recruitment was by email (74%, n = 42). Supplementary Table 1a presents the data on professional recruitment sources and methods.

There was heterogeneity in reporting of patient participant characteristics. Only 10% (n = 8) reported on the patient socio-economic / educational status and only 9% (n = 7) on their ethnicity. Similarly, less than a third of studies reported on either patient experience of the condition, (e.g. length of experience) or an aspect of their treatment experience. Table 5a presents the reporting data on professional characteristics. Additional study design characteristics are presented in Supplementary Table 1b and study characteristics relating to professionals are in Table 1a.

Table 2 around here

Table 3 presents the data on Delphi specific issues, including the duration of rounds, the scoring approaches in round 1 and feedback methods in round 2 (data for subsequent rounds are presented in Supplementary Table 2a) where both patients and professionals scored outcomes. Most studies did not report the duration of their rounds, however, of those that did, the majority reported 2-4 weeks duration per round. The majority of COS studies reported using a 1-9 scoring system (70%, n=52).

Feedback methods were explored for studies reporting more than 1 round. 48 studies reported on which stakeholder groups' feedback was presented to participants, for example, whether patient and professional feedback was presented separately for each group or combined. The most frequent approach was where results for different stakeholder groups were reported separately, (n=21, 44%). A range of feedback types were described by the 43 studies reporting on this, with some studies reporting use of two or more types of feedback. The most common type of feedback was the distribution of scores (65%, n =28); 10 studies (23% of those reporting) described providing either a mean or median only.

Table 3 around here

Table 4 shows the response rates per round. The recruitment sources of the 20 studies where patient response data for round 1 was reported were predominantly treatment centres (45%, n = 9). The median round 1 response rate for patients was 59% compared to 52% for professionals.

The median ratio of professionals to patients was 2.7 (n=61), although some studies reported more than twice as many patients as professionals (e.g. Potter[15]).

Participation rates for rounds 2 and 3 were calculated (excluding studies where non-respondents were invited from previous rounds). The median round 2 response rate for patients was 84% (n = 44), comparable to the professional respondents (median = 85%, n = 46). Response rates in round 3 were the same (91%) for both patients and professionals.

Table 5 explores potential associations between patient response rates, and PPI, Delphi piloting, reminders and methods of recruitment. There is limited reporting of data on these factors with no evidence of an effect of PPI, piloting and reminders on response rates but an indication that recruiting from treatment centres is better in terms of retention in round 2. Round 2 response rates

For peer review only - http://bmjopen.bmj.com/site/about/guidelines.xhtml

for studies recruiting through treatment centres were higher (89%, n =6) than studies recruiting through patient organisations (77%, n = 20) and a combined treatment centre / patient organisation approach (77%, n = 11), although the numbers of studies were small, particularly for those recruiting through the treatment centre.

Discussion

This review has highlighted variations in the design and conduct of COS studies that included patients in the Delphi process, including differing: scoring and feedback systems, approaches to recruiting patients, lengths of time between rounds, and use of reminders, incentives, PPI and piloting. It has also identified potential challenges with the Delphi feedback approaches, minimal reporting of participant characteristics; the lack of translation of Delphi surveys into local languages and indicated that recruitment may be more of a challenge than retention. There were indications that studies which recruited patients through treatment centres had higher round 2 response rates than studies recruiting through patient organisations.

Previous qualitative research, PPI and piloting

Williamson et al[16] recommend using qualitative research or consulting with key stakeholders, including patients, to help identify important outcomes and ensure that the language used to describe outcomes is meaningful for patients. Less than a third of studies used either of these two methods prior to undertaking their Delphi survey. Additionally, Williamson et al[1] suggest that piloting of the Delphi survey can also help the COS development team to refine their outcome labels and explanations, however, only around a third of studies report undertaking piloting. COS developers may be missing opportunities to improve the accessibility of their Delphi surveys. Better reporting of piloting would improve understanding of its impact.

Young & Bagley[8] described the potential benefits that PPI could bring to the COS development process. PPI has the potential, for example, to help with recruitment and retention by improving the accessibility of the study. Less than half of the publications in this review reported undertaking PPI; those that did report PPI provided scant details. It is acknowledged that word restrictions and the journal's focus may limit the amount of space that can be dedicated to discussions about PPI and also that some authors may have chosen to publish separately about PPI in their COS studies, for example, Smith [17]. This review did not include linked papers to the COS studies and this, therefore, limits the conclusions that can be drawn, however, the experience of the COMET Initiative suggests that such detailed publications about PPI in COS and its impact are rare. The few studies that did provide more detailed reports will help future COS developers plan for PPI (e.g. Smith[17] & Crudgington[18]). Improving the reporting of PPI, for example, by following the GRIPP2 checklist[19], would enable the impact of PPI on recruitment and retention to be more accurately investigated.

We explored the potential impact of PPI on patient participation rates, but did not find an association. Minimal reporting of PPI however means that it was also unclear what the quality of PPI was like, for example, one study might have held multiple supported meetings with a number of patients to explore how to define the outcomes for a study, where another study might only have emailed a list of outcomes for feedback from one research partner, with little guidance on how to review the outcomes for a patient audience. Without such detail it is difficult to come to conclusions about the real impact of PPI. Ethnographic work with patient research partners in COS studies will inform our understanding of current PPI practice in this area [20]

Scoring system & feedback

For peer review only - http://bmjopen.bmj.com/site/about/guidelines.xhtml

Our review indicates that the 1-9 scoring system is the most commonly used system in COS studies that include patients, however this scoring system is used in the DelphiManager software, and the large number of electronically delivered studies that reported using this software may, therefore, have influenced this finding. Biggane et al[7] interviewed patients retrospectively about their experience of participating in a Delphi survey, noting that whilst there are statistical considerations influencing the choice of scoring scales, patients can have differing views on the scales used. Whilst some patients in their study preferred the 1-9 scoring scale, others struggled to use it, indicating the need for additional support and guidance. Given the high usage of the 9-point scoring method, further research is warranted to explore how patients and other participants experience, interpret and use this scoring system.

Providing feedback to participants on the scores of other participants in previous rounds is used to drive consensus between stakeholders in Delphi surveys, with stakeholders encouraged to consider the views of others before re-scoring an outcome. A study that compared providing feedback to participants only on the scores of their own peer group, versus providing feedback to participants on the scores from each of the stakeholder groups, found that seeing other groups' perspectives increased consensus[21]. Participants in a study by Fish et al[22]reported "trying to understand the importance of an outcome from the perspective of another participant", as one of the most common reasons for revising their scores between rounds, and this was especially the case for health care professionals. Whilst several studies in our review did not report on their feedback approach, nearly half of those that did report this, did not describe providing feedback to participants by group, instead just presenting feedback from a participant's own stakeholder group or for all participants combined. In the absence of presenting each participant with feedback from each group consensus may not be so easily achieved across stakeholder groups[1]. Of note were two SWAT studies exploring feedback methods, indicating interest in finding the best feedback approach[23, 24]. One of these has been completed, finding that peer feedback reduced variability in scoring compared with combined feedback from multiple groups[23]. It should again be noted that the use of DelphiManager software by a large proportion of studies conducted electronically may have impacted the data on feedback.

In addition to what feedback participants received about the scores of other participants, how feedback was presented also varied in the studies although most presented feedback as a distribution of scores and numerical frequencies. Of studies that reported on how feedback was presented, a fifth described only providing a summary statistic (a median or mean score). This is potentially problematic as there are indications that participants do not understand the term median and that they have issues with fully understanding averages[25]. Fish[25] also found the patients in her study understood and liked seeing the percentage of participants rating each outcome as each of 1 to 9, and yet our review has found that around two thirds of studies did not provide such feedback. Further research is needed to explore the best ways to present feedback so that it is more easily understood.

Patient participation and inclusivity

The COS_STAD (Standards for core outcome set development) specifies that people with lived experience of the condition / intervention should be key stakeholders in the COS development process[26]. Our review explored the ratio of patient participants compared to professionals, finding that patients tended to be in the minority, although there are also examples of COS studies with higher rates of participation amongst patients (e.g. Potter et al[21]). Inclusivity in COS development is crucial to ensure that the outcomes selected in a COS are relevant and important for the diverse range of patients potentially affected by the COS. There have been calls for more

For peer review only - http://bmjopen.bmj.com/site/about/guidelines.xhtml

inclusive research generally, further emphasised by the recent COVID 19 pandemic[27]. In the studies in our review, there was minimal reporting of patient ethnicity and socioeconomic status and the reasons for this warrants further exploration. Additionally, there was minimal reported use of translation meaning that COS completion is restricted to those with the relevant language skills, again limiting its inclusivity.

Given the need to ensure adequate stakeholder diversity and inclusion and the potential impact of attrition (overestimation of consensus if participants with minority perspectives drop out), it is important to explore response rates in all rounds of the COS studies. There are indications that recruiting stakeholder participants into COS studies can be challenging, however, once recruited, retention was quite high for most studies. This echoes findings from Delphi studies in other areas[4]. Retrospective interviews with patient participants in COS Delphi studies have highlighted key areas of concern for them and provided some initial insights on their motivation to participate[7]. However, further research is needed that explores patients' motivation to take part soon after the recruitment decision to inform the development of future recruitment resources.

Associations with patient participation rates

We aimed to explore how study characteristics such as PPI, piloting, reminders, recruitment methods and sources influenced the participation of patients. The reporting of recruitment in the reviewed studies was complex and sometimes unclear. Our comparison of recruitment sources and response rates was limited due to problems with reporting. However, studies using treatment centres as a source for recruitment appeared to have higher round 2 response rates. This echoes previous findings [25] indicating lower attrition amongst patient participants recruited via treatment centres compared to those recruited through patient organisations and social media. This warrants further research.

Study limitations and future research

This study is limited by omissions in reporting about the design and delivery of studies. Recent guidance about COS development and reporting[28] and guidance on PPI reporting[19] may improve the description of COS studies in the future. We are planning to interview COS developers to explore their perspectives on the design of COS Delphi studies, including the use of patient facing resources to recruit and retain patients in a Delphi survey and materials to support their participation. We will work closely with a PPI panel to review these materials, alongside the findings of this current review and the future findings from interviews with COS developers, to enhance the accessibility, ease of use and appeal of the materials.

Conclusion

This study has explored the participation of patients in COS studies. Variability was striking in how COS Delphi surveys were designed and conducted to include patient participants and other stakeholders. Future research would be useful to explore what motivates patients to take part in COS studies and what factors influence recruitment strategies used by COS developers. Reporting needs to be improved to increase knowledge of how methods affect patient participation, in particular reporting response rates and denominators for all rounds by stakeholder group, more detailed descriptions of PPI, piloting, recruitment methods and sources.

References

For peer review only - http://bmjopen.bmj.com/site/about/guidelines.xhtml

1. Williamson, P., et al., *The COMET Handbook: version 1.0*. 2017: BioMed Central.
2. Berglas, S., et al., *Patients' perspectives can be integrated in health technology assessments: An exploratory analysis of CADTH common drug review.* Research Involvement and Engagement, 2016. **2**(1).
3. Gargon, E., S.L. Gorst, and P.R. Williamson, *Choosing important health outcomes for comparative effectiveness research: 5th annual update to a systematic review of core outcome sets for research.* PLoS ONE, 2019. **14**(12): p. 1-18.
4. Keeney, S., F. Hasson, and M. H, *The Delphi Technique in Nursing and Health Research*. 2010: Wiley, Blackwell.
5. Gargon, E., et al., *Choosing important health outcomes for comparative effectiveness research: 6th annual update to a systematic review of core outcome sets for research.* PLoS ONE, 2021. **16**(1): p. 1-12.
6. Gargon, E., P.R. Williamson, and B. Young, *Improving core outcome set development: qualitative interviews with developers provided pointers to inform guidance.* Journal of Clinical Epidemiology, 2017. **86**: p. 140-152.
7. Biggane, A.M., et al., *Participating in core outcome set development via Delphi surveys: qualitative interviews provide pointers to inform guidance.* BMJ open, 2019. **9**(11): p. e032338.
8. Young, B. and H. Bagley, *Including patients in core outcome set development: issues to consider based on three workshops with around 100 international delegates.* Research Involvement and Engagement, 2016. **2**(1).
9. Gargon, E., et al., *Choosing Important Health Outcomes for Comparative Effectiveness Research: A Systematic Review.* PLoS ONE, 2014. **9**(6): p. 1-12.
10. Gorst, S.L., et al., *Choosing Important Health Outcomes for Comparative Effectiveness Research: An Updated Review and User Survey.* PLoS ONE, 2016. **11**(1): p. 1-12.
11. Gorst, S.L., et al., *Choosing important health outcomes for comparative effectiveness research: An updated review and identification of gaps.* PLoS ONE, 2016. **11**(12).
12. Davis, K., et al., *Choosing important health outcomes for comparative effectiveness research: An updated systematic review and involvement of low and middle income countries.* PLoS ONE, 2018. **13**(2): p. 1-14.
13. Gargon, E., et al., *Choosing important health outcomes for comparative effectiveness research: 4th annual update to a systematic review of core outcome sets for research.* PLoS ONE, 2018. **13**(12): p. 1-15.
14. Hall, D., et al., *The COMiT'ID Study: Developing Core Outcome Domains Sets for Clinical Trials of Sound-, Psychology-, and Pharmacology-Based Interventions for Chronic Subjective Tinnitus in Adults.* Trends in Hearing, 2018. **22**.
15. Potter, S., et al., *Development of a core outcome set for research and audit studies in reconstructive breast surgery.* British Journal of Surgery, 2015. **102**(11): p. 1360.
16. Williamson, P.R., et al., *Controversy and Debate Series on Core Outcome Sets. Paper 4: Debate on Paper 1 from the perspective of COMET [Core Outcome Measures in Effectiveness Trials]*. 2020. p. 222-224.
17. Smith, H., et al., *Defining and evaluating novel procedures for involving patients in Core Outcome Set research: creating a meaningful long list of candidate outcome domains.* Research Involvement and Engagement, 2018. **4**(1): p. 1-12.
18. Crudgington, H., et al. *Core Health Outcomes in Childhood Epilepsy (CHOICE): Development of a core outcome set using systematic review methods and a Delphi survey consensus*. 2019. Netherlands: John Wiley & Sons Ltd.
19. Staniszewska, S., et al., *GRIPP2 reporting checklists: tools to improve reporting of patient and public involvement in research.* Research Involvement and Engagement, 2017. **3**(1): p. 1-11.

For peer review only - http://bmjopen.bmj.com/site/about/guidelines.xhtml

20. Brading L M, V.A., Bagley H J, Williamson P R, Woolfall K & Young B., *Distinctive challenges of patient and publicinvolvement in core outcome set development:qualitative study - Contributed Talks.* Journal of Evidence Based Medicine, 2019. **12**(S1): p. 5-31.
21. Brookes, S.T., et al., *Three nested randomized controlled trials of peer-only or multiple stakeholder group feedback within Delphi surveys during core outcome and information set development.* Trials, 2016. **17**(1).
22. Fish, R., et al., *"Vicarious thinking" was a key driver of score change in Delphi surveys for COS development and is facilitated by feedback of results.* Journal of Clinical Epidemiology, 2020. **128**: p. 118-129.
23. MacLennan, S., et al., *A randomized trial comparing three Delphi feedback strategies found no evidence of a difference in a setting with high initial agreement.* Journal of Clinical Epidemiology, 2018. **93**: p. 1-8.
24. Blackwood, B., et al., *Core Outcomes in Ventilation Trials (COVenT): protocol for a core outcome set using a Delphi survey with a nested randomised trial and observational cohort study*. 2015, Springer Nature.
25. Fish, R., *Development of a core outcome set for trials of chemoradiotherapy for anal squamous cell carcinoma*. 2018, University of Manchester.
26. Kirkham, J.J., et al., *Core Outcome Set-STAndards for Development: The COS-STAD recommendations.* PLoS Medicine, 2017. **14**(11): p. e1002447-e1002447.
27. Miles, D.W., et al., *Developing a roadmap to improve trial delivery for under-served groups: results from a UK multi-stakeholder process.* Trials, 2020. **21**(1): p. 1-9.
28. Kirkham, J.J., et al., *Core Outcome Set-STAndards for Reporting: The COS-STAR Statement.* PLoS Medicine, 2016. **13**(10): p. e1002148-e1002148.

Footnotes

Twitter @COMETinitiative

Author statement

Contributors: Conceptualization: HB, PRW, BY; Funding acquisition: PRW; Investigation: HB, PRW, BY; Methodology: HB, PRW, BY; Writing – original draft: HB; Writing – review & editing: HB, PRW, BY

Funding: HB is supported by the National Institutes for Health Research (NIHR) through award number NF-SI_0513-10025. The views expressed in this article are those of the author(s) and not necessarily those of the NIHR, or the Department of Health and Social Care. PW is also supported by the Medical Research Council (MRC) Trials Methodology Research Partnership (grant reference MR/S014357/1).

Conflicts of interests: PW & HB are members of the COMET Management Group, BY and HB are members of the COMET PoPPIE Working Group.

Ethics approval: This systematic review was based on published data. Therefore, obtaining ethical approval was unnecessary.

Patient consent for publication: Not applicable

Data availability statement: On reasonable request from the first author

Tables

For peer review only

For peer review only - http://bmjopen.bmj.com/site/about/guidelines.xhtml

Table 1 – Scope of the Core Outcome Set

Core Outcome Set Scope	
**Health area**	**n (%)**
Anaesthesia & pain control	1 (1%)
Blood disorders	1 (1%)
Cancer	9 (12%)
Cancer/ Child health	1 (1%)
Child health	1 (1%)
Child health/ Ear, nose & throat	1 (1%)
Child health/ Gastroenterology	1 (1%)
Ear, nose & throat	4 (5%)[a]
Endocrine & metabolic	3 (4%)
Eyes & Vision	1 (1%)
Gastroenterology	6 (8%)
Healthcare of older people	2 (3%)
Heart & circulation	3 (4%)
Heart & Circulation and skin	3 (4%)[a]
Kidney disease	2 (3%)
Lungs & airways	2 (3%)
Mental health	1 (1%)
Neonatal care	1 (1%)
Neurology	4 (5%)[b]
Neurology / eyes & vision	1 (1%)
Orthopaedics & trauma	6 (8%)
Other	2 (3%)
Overweight / obesity	1 (1%)
Pregnancy & childbirth	11 (14%)[c]
Rehabilitation	1 (1%)
Rehabilitation, Rheumatology	1 (1%)
Rheumatology	2 (3%)
Skin	4 (5%)
Tobacco, drugs & alcohol dependence	1 (1%)
**Adults / Children**	**n (%)**
Adults	45 (58%)
Both adults and children	18 (23%)
Children	11 (14%)
Not reported	4 (5%)
**Gender**	**n (%)**
Male only	2 (3%)
Female only	8 (10%)
Both	68 (87%)
**Intervention**	**n (%)**
Any	49 (63%)
Drug	4 (5%)
Psychological	3 (4%)
Surgery	7 (9%)
Other[d]	15 (19%)
**Countries (all participants)**	**n (%)**

For peer review only - http://bmjopen.bmj.com/site/about/guidelines.xhtml

1 only	11 (19%)
2-10	14 (24%)
11-10	8 (14%)
20-30	13 (22%)
>30	13 (22%)
Not reported / unclear	19

Footnotes:

[a] Includes articles reporting 3 COS studies

[b]Includes articles reporting 2 COS studies

[c] Includes 2 articles reporting 2 COS studies

[d]Other: active surveillance anaesthetic techniques; behavioural; chemoradiotherapy; ECMO; gene therapy; haemodialysis; health care transition; interdisciplinary multimodal pain therapy; medication review; physical activity intervention; pre-pregnancy care; procedure (induction of labour); rehabilitation; sound-based interventions; visual screening / assessment.

Table 2 - Study characteristics of the Delphi studies

Study characteristics	
**Methods to explore patients' views on important outcomes prior to the Delphi study[a]**	**n (%)**
Patient interviews	20 (26%)
Survey	12 (12%)[b]
Nominal group technique	3(4%)
Focus groups	4 (5%)
Not reported / unclear	47
**Pilot Delphi undertaken**	**n (%)**
Pilot study reported	28 (36%)[c]
**Patient and Public Involvement (PPI)**	**n (%)**
PPI reported	31 (40%)
**Method of delivery (LE)**	**n (%)**
Electronic	39 (74%)
Post	4 (8%)
Face to face	3 (6%)
Mixture of approaches	7 (13%)
Not reported	19
Unclear	6
**Reminders**	**n (%)**
1 reminder between rounds	10 (31%)
More than one reminder between rounds	22 (69%)
Reminders sent but number of reminders not reported	12
Not reported	46[d]
**Incentives (patient participants)**	**n (%)**
Yes (monetary incentive / voucher)	3 (38%)

For peer review only - http://bmjopen.bmj.com/site/about/guidelines.xhtml

Yes (non-monetary incentive)[e]	3 (38%)
Incentive not offered	2 (25%)
Not reported	70
**Language used with patients**	**n (%)**
Translation	10 (20%)
Conducted in English (specifically stated)	19 (37%)
Native language (implicit)	22 (43%)
Not reported	27
**Participant recruitment source & approach[f]**	
Recruitment source (patients)	n (%)
Patient organisation	43 (62%)
Clinic / Treatment centre	31 (45%)
Social media	19 (28%)
PPI group (external to the COS study)	14 (20%)
Contacts of Steering Committee / Management Group	7 (10%)
Snowball sampling	10 (15%)
Research database	6 (9%)
Other[g]	See footnote
Unclear[h]	3
Not reported	6
**Recruitment approach (patients)**	**n (%)**
Email invitation	42 (74%)
Postal invitation	5 (9%)
Telephone invitation	4 (7%)
Information provided in clinic	7 (12%)
Poster / newsletter	7 (12%)
e-source (website / social media)	15 (30%)
Recruitment approach unclear	5
Not reported	16
**Participant characteristics reported**	
**Patient participants**	**n (%)**
Age	39 (50%)[i]
Gender	44 (56%)[j]
Socio-economic / education	8 (10%)[k]
Ethnicity	8 (10%)[l]
Marital status	7 (9%)
Experience of condition	24 (31%)
Experience of treatment	15 (19%)
Other[m]	See footnote

Footnotes:

[a]Some studies used more than one approach to explore patients views on outcomes prior to the Delphi.

[b]Including 6 studies in which patients identified outcomes in what the authors referred to as ‘round 1’.

[c]Including 3 studies where pilots were without patients

For peer review only - http://bmjopen.bmj.com/site/about/guidelines.xhtml

[d]Including 12studies where reminders were sent but the number of reminders was not reported

[e]All non-monetary were certificates and reported in a single article

[f]More than one recruitment source / approach may have been used.

[g]Other included through a professional organisation (n=2), a conference attended by patients (n=3, 3 COS from the same article), previous participation in a research study (n=4) and participating researchers identified patients (n=1)

[h]Additional articles partially unclear, recruitment source (n=3), recruitment approach (n=3)

[i]Including 5 studies where age was reported collectively for both patients and professionals and 1 study where age reported for parent's child only

[j]Including 12 where COS study was specifically targeted at one gender and 9 studies where gender was reported collectively for both patients and professionals.

[k]Including 1 study where education was reported collectively for both patients and professionals

[l]Including 2 studies where ethnicity was reported collectively for both patients and professionals.

[m]Other- previous participation in research (n=2, both of which reported collectively for both patients and professionals), number of children (n=1), home type (n = 1)

Table 3 – Delphi specific survey issues

**Duration of rounds**				
**Round duration**	**n (%)**			
**Time for each round**	**< 2 weeks**	**2– 4 weeks**	**>4 weeks**	**Not reported / not clear / n/a**
Round 1	1 (3%)	23 (70%)	9 (27%)	45
Round 2	1 (3%)	25 (78%)	6(19%)	46
Round 3	0	16 (80%)	4 (20%)	58
**Scoring Systems and Feedback Approaches**				
**Scoring system (Round 1)**				**n (%)**
1-9 / 1-10[a]				52 (70%)[b]
0-4/1-4 / 1-5				12 (16%)
9/10/12 most important outcomes				4 (5%)
Yes/no/don't know or agree/disagree/unsure				7 (9%)
Not reported				2
Unclear				1
**Source of stakeholder feedback Round 2**				**n (%)**
All stakeholder groups combined				10[c] (21%)
Stakeholder groups reported separately				21 (44%)
Own Stakeholder group only				10[d] (21%)
Stakeholder groups reported separately and all stakeholder groups combined				5 (10%)
SWAT[e] – different groups saw different feedback				1 (4%)
N/a patients only took part in 1 round				13
Not reported				13
Unclear				4

For peer review only - http://bmjopen.bmj.com/site/about/guidelines.xhtml

Feedback type reported[f]	n (%)
Graphical feedback[g]	17 (40%)
Numerical frequencies	24 (56%)
Summary statistics[g]	15 (35%)[h]
Dispersion / distribution of scores	28 (65%)
Anonymised comments from prior round	2 (5%)
N/a patients only voted in one round	13
Not reported	22

Footnotes

[a]Only two studies used 1-10

[b]Children in one of these studies used 1-3 scale and Caregivers in another study scored differently to patients in one of these studies – patients used score cards

[c]Including one study which also provided the patient group scores and one study in which participants could request feedback by stakeholder group

[d]Including one study which also provided combined scores for all

[e]SWAT – Study Within a Trial

[f]Studies could report more than one type of feedback

[g]Excludes anywhere it was unclear whether the feedback type was reported

[h]10 studies reported only summary statistics

Table 4 – Response rates

Round	Participation[a]	Median, Min, Max
1	Patients invited and completed (n=20)	59%, 11%, 95%
	Professionals invited and completed (n = 20)	52%, 19%, 93%
	Ratio of Professionals to patients (n=62)	2.7, 4.1, 0.4, 23
2	Patients invited and completed (n=44)	84%, 32%, 100%
	Professionals invited and completed (n=46)	85%, 43%, 100%
3	Patients invited and completed (n=20)	91%, 50%, 100%
	Professionals invited and completed (n=24)	91%, 78%, 100%

[a] In round 2 and / or round 3 some studies described non-responders to a previous round being invited into the round (this could be both patient and professional previous responders or just one type of previous responder). These studies were excluded from analysis of round 2 and / or round 3 response rate data for the relevant category of respondent. Round 1 participation rates were available for studies where the denominator was known (i.e. the number of people invited).

For peer review only - http://bmjopen.bmj.com/site/about/guidelines.xhtml

Table 5 – Association between patient response rate and PPI, piloting and recruitment source

Factor	Round[a]	Factor category	Patients- median response rate, min, max
**PPI**	**1**	PPI (n=6)	62%, 36%, 77%
		PPI not reported (n=14)	59%, 11%, 95%
	**2**	PPI (n=22)	78%, 32%, 94%
		PPI not reported (n = 22)	86%, 50%, 100%
	**3**	PPI (n=9)	92%, 71%, 100%
		PPI not reported (n =11)	90%, 50%, 100%
**Piloting**	1	Piloting (n = 10)	61%, 36%, 95%
		No piloting reported (n=10)	58%, 11%, 91%
	2	Piloting (n =21)	84%, 41%, 100%
		No piloting reported (n = 23)	83%, 32%, 100%
	3	Piloting (n =9)	92%, 71%, 100%
		No piloting reported (n=11)	89%, 50%, 100%
**Recruitment source**	2	Treatment Centre (n=6)	89%, 83%, 90%
		Patient organisation (n = 20)	77%, 32%, 100%
		Treatment centre and patient organisation (n = 11)	77%, 50%, 93%
		Neither treatment centre nor patient organisation (n = 5)	94%, 90%, 100%
		Nothing reported on recruitment source (n = 2)	92%, 84%, 100%
**Reminders**	2	Reminders (n = 30)	82, 32,96
		No reminders reported (n = 14)	86, 57, 100

Footnote

[a]19 studies with round 1 data on participation rate, 44 studies with round 2 completion rate and 20 with round 3 completion rate data.

For peer review only - http://bmjopen.bmj.com/site/about/guidelines.xhtml

Patient participation in Delphi surveys to develop core outcome sets: systematic review

Authors: Barrington H.J.[1], Young B.[1] & Williamson P.R.[1]

Author affiliations: [1]University of Liverpool, Liverpool, U.K.

Supplementary tables

Table 1a – Study characteristics (professional participants)

**Professional recruitment source & approach[a]**	
**Professional recruitment source**	**n (%)**
Professional organisation	49 (70%)
Publication authors (including Cochrane authors)	22 (31%)
Research study	13(19%)
Research group / consortium / CTU groups (including Cochrane group)	32 (46%)
Steering group members / contacts / University contacts	14 (20%)
Treatment centres	15 (21%)
Snowball sampling	25 (36%)
Other[b]	See below
Not reported	8
**Professional recruitment approach**	**n (%)**
Email invitation	50 (91%)
Postal invitation	4 (7%)
Handed invitation	4 (7%)
Newsletter / webpage	5 (9%)
Unclear	3
Not reported	20
**Participant characteristics reported**	
**Professional participants**	**n (%)**
Clinical experience	20 (26%)
Research experience	9 (12%)[c]
Gender	24 (31%)[d]
Age	21 (27%)e
Ethnicity	4 (5%)c
Education	3 (4%)f

Footnotes

[a] More than one recruitment source could be used

[b] Other included journal editorial groups (9), through informal mailing lists (n=2), members of steering committee (n=2), conference / conference special interest group (n=4) email discussion group / special interest group (n=4), research funding organisation (n = 2), audit participant (n=1)

[c]Includes 2 studies where characteristic reported collectively on research experience and ethnicity for PE and LE

[d]Includes 9 studies where characteristic reported collectively on gender for professionals and patients

[e]Includes 5 studies where characteristic reported collectively for professionals and patients

[f]Includes 1 study where characteristic reported collectively for professionals and patients

Table 1b - Study development and design characteristics of the Delphi studies

Study design & development characteristics	
**Number of rounds where patients participated**	**n (%)**
1	13 (17%)
2	28 (36%)
3	37 (47%)
**Number of stakeholder participant categories**	**n (%)**
2	31 (40%)
3	20 (26%)
4	16 (21%)
5	10 (13%)
6	1 (1%)
**Number of reported items per round**	**Descriptive statistics[a]**
Round 1 (n=71)	Median = 46, Min = 9, Max = 130
Round 2 (n=53)	Median = 49, Min = 8, Max = 130
Round 3 (n=28)	Median = 37, Min = 7, Max = 114

Footnote

[a] excluding not reported, n/a, unclear

Table 2a – Delphi characteristics rounds 2 and 3

Scoring System Rounds 2 &3		
**Scoring system**	**Round 2 n (%)**	**Round 3 n (%)**
1-9 / 1-10[a]	52 (85%)	26 (77%)
0-4/1-4 / 1-5	4 (7%)	3 (9%)
9/10/12 most important outcomes	2 (3%)[b]	1 (3%)
Yes/no/don't know or agree/disagree/unsure	2 (3%)	1 (3%)
Yes/no/include in COS & Essential and recommended outcomes	n/a	3 (9%)
Domain inner core, middle ring, outer ring	1 (2%)	n/a
Not reported	2	1
Unclear	2	2
n/a patients only in 1 round	13	13
n/a only 2 rounds	0	28
**Feedback**		
**Feedback type Round 3**		**n (%)**
All stakeholder groups combined		7 (28%)

For peer review only - http://bmjopen.bmj.com/site/about/guidelines.xhtml

Stakeholder groups reported separately	9 (36%)
Own stakeholder group	1 (4%)
Each stakeholder group & all stakeholder groups combined	3 (12%)
Own stakeholder group & all stakeholder groups combined	3 (12%)
SWAT	2 (8%)
Not reported	6
N/a only 2 rounds	28
N/a patients only took part in one round	13
Unclear	6

Footnotes

[a] Only two studies used 1-10

[b] Caregivers scored differently to patients in one of these studies – patients used score cards

For peer review only

For peer review only - http://bmjopen.bmj.com/site/about/guidelines.xhtml

Supplementary Figure 1 - Preferred Reporting Items for Systematic Reviews and Meta-Analyses flowchart of identification of eligible studies from the COMET database. Data were extracted from the COS systematic reviews

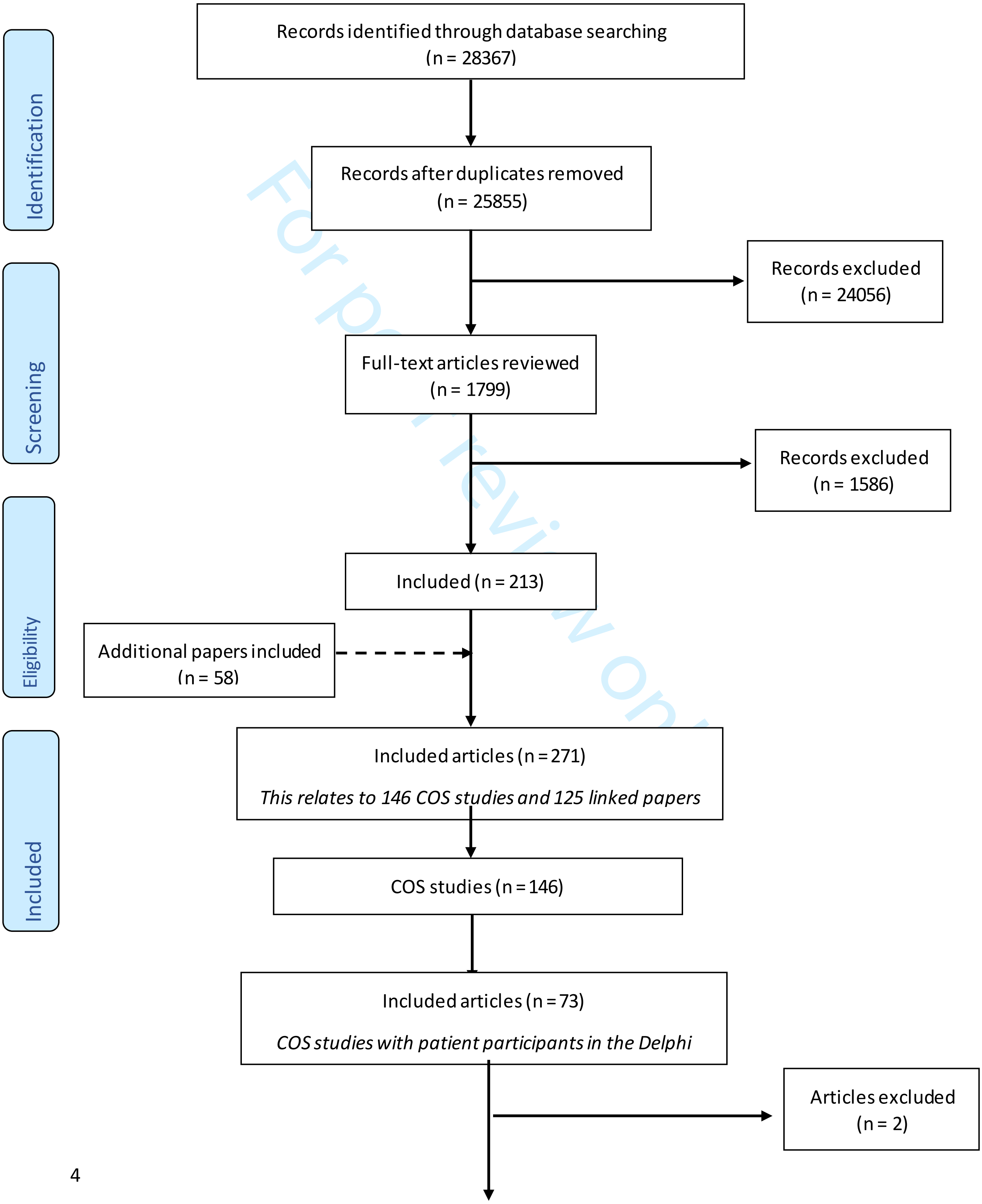

For peer review only - http://bmjopen.bmj.com/site/about/guidelines.xhtml

Included articles (n = 71)

COS studies with more than one patient participant in the Delphi

5

For peer review only

Reporting checklist for systematic review and meta-analysis.

Based on the PRISMA guidelines.

Instructions to authors

Complete this checklist by entering the page numbers from your manuscript where readers will find each of the items listed below.

Your article may not currently address all the items on the checklist. Please modify your text to include the missing information. If you are certain that an item does not apply, please write "n/a" and provide a short explanation.

Upload your completed checklist as an extra file when you submit to a journal.

In your methods section, say that you used the PRISMAreporting guidelines, and cite them as:

Moher D, Liberati A, Tetzlaff J, Altman DG, The PRISMA Group. Preferred Reporting Items for Systematic Reviews and Meta-Analyses: The PRISMA Statement

For peer review only

		Reporting Item	Page Number
**Title**			
	#1	Identify the report as a systematic review, meta-analysis, or both.	1
**Abstract**			

Structured summary	#2	Provide a structured summary including, as applicable: background; objectives; data sources; study eligibility criteria, participants, and interventions; study appraisal and synthesis methods; results; limitations; conclusions and implications of key findings; systematic review registration number	1
**Introduction**			
Rationale	#3	Describe the rationale for the review in the context of what is already known.	2
Objectives	#4	Provide an explicit statement of questions being addressed with reference to participants, interventions, comparisons, outcomes, and study design (PICOS).	2
**Methods**			
Protocol and registration	#5	Indicate if a review protocol exists, if and where it can be accessed (e.g., Web address) and, if available, provide registration information including the registration number.	3
Eligibility criteria	#6	Specify study characteristics (e.g., PICOS, length of follow-up) and report characteristics (e.g., years considered, language, publication status) used as criteria for eligibility, giving rational	3
Information sources	#7	Describe all information sources in the search (e.g., databases with dates of coverage, contact with study authors to identify additional studies) and date last searched.	3

For peer review only

Search	#8	Present full electronic search strategy for at least one database, including any limits used, such that it could be repeated.	3
Study selection	#9	State the process for selecting studies (i.e., for screening, for determining eligibility, for inclusion in the systematic review, and, if applicable, for inclusion in the meta-analysis).	3
Data collection process	#10	Describe the method of data extraction from reports (e.g., piloted forms, independently by two reviewers) and any processes for obtaining and confirming data from investigators.	3
Data items	#11	List and define all variables for which data were sought (e.g., PICOS, funding sources), and any assumptions and simplifications made.	3
Risk of bias in individual studies	#12	Describe methods used for assessing risk of bias in individual studies (including specification of whether this was done at the study or outcome level, or both), and how this information is to be used in any data synthesis.	N/A
Summary measures	#13	State the principal summary measures (e.g., risk ratio, difference in means).	3/4
Planned methods of analyis	#14	Describe the methods of handling data and combining results of studies, if done, including measures of consistency (e.g., I2) for each meta-analysis.	3/4

For peer review only

Risk of bias across studies	#15	Specify any assessment of risk of bias that may affect the cumulative evidence (e.g., publication bias, selective reporting within studies).	N/A
Additional analyses	#16	Describe methods of additional analyses (e.g., sensitivity or subgroup analyses, meta-regression), if done, indicating which were pre-specified.	N/A

Results

Study selection	#17	Give numbers of studies screened, assessed for eligibility, and included in the review, with reasons for exclusions at each stage, ideally with a flow diagram.	4
Study characteristics	#18	For each study, present characteristics for which data were extracted (e.g., study size, PICOS, follow-up period) and provide the citation.	4
Risk of bias within studies	#19	Present data on risk of bias of each study and, if available, any outcome-level assessment (see Item 12).	N/A
Results of individual studies	#20	For all outcomes considered (benefits and harms), present, for each study: (a) simple summary data for each intervention group and (b) effect estimates and confidence intervals, ideally with a forest plot.	N/A
Synthesis of results	#21	Present the main results of the review. If meta-analyses are done, include for each, confidence intervals and measures of consistency.	4

For peer review only

Risk of bias across studies	#22	Present results of any assessment of risk of bias across studies (see Item 15).	N/A
Additional analysis	#23	Give results of additional analyses, if done (e.g., sensitivity or subgroup analyses, meta-regression [see Item 16]).	N/A
**Discussion**			
Summary of Evidence	#24	Summarize the main findings, including the strength of evidence for each main outcome; consider their relevance to key groups (e.g., health care providers, users, and policy makers	6-8
Limitations	#25	Discuss limitations at study and outcome level (e.g., risk of bias), and at review level (e.g., incomplete retrieval of identified research, reporting bias).	6-8
Conclusions	#26	Provide a general interpretation of the results in the context of other evidence, and implications for future research.	8
**Funding**			
Funding	#27	Describe sources of funding or other support (e.g., supply of data) for the systematic review; role of funders for the systematic review.	9

None The PRISMA checklist is distributed under the terms of the Creative Commons Attribution License CC-BY. This checklist can be completed online using https://www.goodreports.org/, a tool made by the EQUATOR Network in collaboration with Penelope.ai

For peer review only
